# HIV-associated immune remodeling in cervical cancer: implications for immunotherapy, biomarkers, and trial design

**DOI:** 10.3389/fimmu.2026.1953030

**Published:** 2026-09-14

**Authors:** Li Huang, Yi Wan, Zhou Yang, Zhuo Zhou, Manna Zhang

**Affiliations:** 1 Department of Oncology, Guiyang Public Health Clinical Center, Guiyang, Guizhou, China; 2 Department of Infectious Diseases, Guiyang Public Health Clinical Center, Guiyang, Guizhou, China; 3 Department of Obstetrics and Gynecology, Guiyang Public Health Clinical Center, Guiyang, Guizhou, China; 4 Department of Pathology, Guiyang Public Health Clinical Center, Guiyang, Guizhou, China

**Keywords:** biomarkers, cervical cancer, HIV, human papillomavirus, immune checkpoint inhibitors, mucosal immunity, T-cell exhaustion, tumor immune microenvironment

## Abstract

Women living with HIV experience a disproportionate burden of persistent high-risk human papillomavirus infection and cervical cancer, yet they remain underrepresented in pivotal immunotherapy trials. Consequently, the effects of HIV-associated immune remodeling on the cervical cancer tumor immune microenvironment, biomarker interpretation, and response to immune checkpoint inhibition remain poorly defined. In this Review, we synthesize evidence concerning chronic inflammation, T-cell dysfunction and exhaustion, impaired antigen presentation, myeloid-mediated immunosuppression, and mucosal and microbial disruption in cervical cancer among women living with HIV. Because direct evidence in this population remains limited, we distinguish findings derived from HIV-associated cervical cancer from supporting evidence obtained in other HIV-associated malignancies, HIV-negative cervical cancer, and broader studies of HIV, HPV, and mucosal immunology. We propose a provisional inflamed, exhausted, and mucosally disrupted (IEM) framework to organize these interacting immune processes and generate hypotheses for prospective validation. This framework highlights why conventional biomarkers, including PD-L1 expression and peripheral CD4 T-cell counts, may incompletely capture immunotherapy-relevant immune heterogeneity and supports the evaluation of multidimensional biomarkers. We further discuss how the proposed framework could inform mechanism-matched therapeutic hypotheses and a novel two-dimensional clinical trial stratification model, while explicitly emphasizing that IEM is not yet a validated clinical phenotype or routine treatment-selection tool. Prospective studies incorporating women living with HIV are required to determine whether these immune features predict immunotherapy efficacy or toxicity and to establish more inclusive, biologically informed clinical-trial strategies.

## 1 Introduction: why HIV-associated cervical cancer demands a distinct immunotherapeutic framework

The integration of immune checkpoint inhibitors (ICIs) into the management of cervical cancer represents one of the most consequential therapeutic advances in gynecologic oncology of the past decade. Phase III trials including KEYNOTE-826 (1), EMPOWER-Cervical 1 (2), BEATcc (3), and KEYNOTE-A18 (4) have collectively reshaped both frontline and locally advanced disease management, delivering clinically meaningful improvements in progression-free and overall survival. The biological foundation for this success is robust: cervical cancer is essentially a virally driven malignancy, with more than 95% of cases attributable to persistent high-risk HPV (hrHPV) infection (5, 6). The constitutive expression of HPV E6 and E7 oncoproteins generates a stable repertoire of viral tumor antigens, rendering cervical tumors highly immunogenic and theoretically tractable solid cancers and providing a clear mechanistic rationale for both checkpoint blockade and therapeutic HPV vaccination (7, 8).

However, the clinical translation of these advances has unmasked a stark and persistent global disparity. Cervical cancer remains the fourth most commonly diagnosed cancer and the fourth leading cause of cancer-related death among women worldwide, with an estimated 660, 000 new cases and 350, 000 deaths annually (9). Crucially, this burden is not distributed equitably. Women living with HIV (WLWH) have an approximately six-fold higher incidence of cervical cancer than HIV-negative women (10), develop invasive disease nearly a decade earlier (11), and account for a disproportionate share of cases in sub-Saharan Africa, where the dual epidemics of HIV and HPV intersect with under-resourced screening and treatment infrastructures (12, 13). Cervical cancer remains an AIDS-defining malignancy and has, in several high-prevalence settings, become the leading cause of cancer mortality in women (13).

Despite this epidemiological reality, WLWH have been systematically excluded from—or severely underrepresented in—the pivotal ICI trials that established current standards of care (14, 15). Protocol-level review of selected immunotherapy trials (**Table 1**) reveals that “known HIV infection” was historically a near-universal exclusion criterion and that, even in trials permitting virologically suppressed participants, HIV-specific subgroup outcomes were rarely prespecified or reported (15). As a consequence, the evidence base guiding the treatment of metastatic and recurrent cervical cancer in WLWH is built almost entirely on inference from HIV-negative populations—an extrapolation whose biological validity is increasingly difficult to defend (**Figure 1B**).

**Table 1:** HIV-related eligibility and representation in selected clinical trials of immune checkpoint inhibitors for cervical cancer and solid tumors.

Trial (NCT)	Phase/setting	Regimen	HIV-related eligibility criteria	PLWH enrolled/reported	Selected efficacy outcome	Implication for WLWH
KEYNOTE-158 (NCT02628067)	Phase II; previously treated recurrent/metastatic	Pembrolizumab monotherapy	Excluded known HIV infection per protocol	None reported	ORR ~14% in PD-L1+ tumors	Direct evidentiary value severely limited; no HIV-specific subgroup
KEYNOTE-826 (NCT03635567)	Phase III; first-line persistent/recurrent/metastatic	Pembrolizumab + platinum-based chemotherapy ± bevacizumab	Excluded active HIV; HIV+ on ART with undetectable viral load and CD4 ≥350 cells/μL permitted per protocol amendment	Not separately reported in primary or final analyses	OS HR 0.63 (95% CI 0.50–0.81) in PD-L1 CPS ≥1 population	Pivotal evidence base does not include disaggregated WLWH outcomes
EMPOWER-Cervical 1 (NCT03257267)	Phase III; second-line post-platinum recurrent/metastatic	Cemiplimab monotherapy vs investigator’s choice chemotherapy	Excluded known HIV infection	None reported	OS HR 0.69 (95% CI 0.56–0.84)	Direct extrapolation to WLWH is unsupported
BEATcc (NCT03556839)	Phase III; first-line metastatic/recurrent/persistent	Atezolizumab + bevacizumab + platinum-paclitaxel	Excluded known HIV infection	None reported	OS HR 0.68 (95% CI 0.52–0.88)	WLWH-specific safety with quadruplet regimen unknown
KEYNOTE-A18 (NCT04221945)	Phase III; locally advanced (FIGO 2014 IB2–IVA)	Pembrolizumab + chemoradiotherapy	Excluded active HIV; clinically stable HIV+ permitted under amendment	Not separately reported	PFS HR 0.70 (95% CI 0.55–0.89)	Immunotherapy–CRT combination data in WLWH absent despite high disease burden
CALLA (NCT03830866)	Phase III; locally advanced	Durvalumab + chemoradiotherapy	Excluded known HIV infection	None reported	PFS HR 0.84 (95% CI 0.65–1.08); did not meet primary endpoint	Neither efficacy nor safety with CRT-ICI in WLWH established
CheckMate 358 (NCT02488759)	Phase I/II; recurrent/metastatic HPV-associated cancers	Nivolumab ± ipilimumab	Excluded known HIV infection (except for exploratory virus-associated sub-cohort)	Small exploratory HIV+ sub-cohort	ORR 26% (nivolumab monotherapy, cervical cohort)	Provides early biological proof-of-concept; underpowered for definitive efficacy
AMC-095/CITN-12 (NCT02408861)	Phase I; HIV+ patients with advanced solid tumors (incl. cervical)	Pembrolizumab monotherapy	Required documented HIV; stratified by CD4 count	n=30 (across tumor types; small cervical subset)	Acceptable safety; no unexpected HIV-related toxicity	Sole prospective ICI safety dataset in PLWH; insufficient to guide cervical-specific practice

HIV-related eligibility criteria were derived from publicly available trial protocols and amendments. “Not separately reported” indicates that HIV-specific enrollment or outcomes were not disaggregated in the cited primary or final reports. The efficacy results shown are selected trial-level outcomes and should not be used for cross-trial comparisons.

ART, antiretroviral therapy; CD4, cluster of differentiation 4; CI, confidence interval; CPS, combined positive score; CRT, chemoradiotherapy; FIGO, International Federation of Gynecology and Obstetrics; HIV, human immunodeficiency virus; HPV, human papillomavirus; HR, hazard ratio; ICI, immune checkpoint inhibitor; ITT, intention-to-treat; n, number of participants; NCT, ClinicalTrials.gov identifier; ORR, objective response rate; OS, overall survival; PD-L1, programmed death-ligand 1; PFS, progression-free survival; PLWH, people living with HIV; WLWH, women living with HIV.

**Figure 1:**
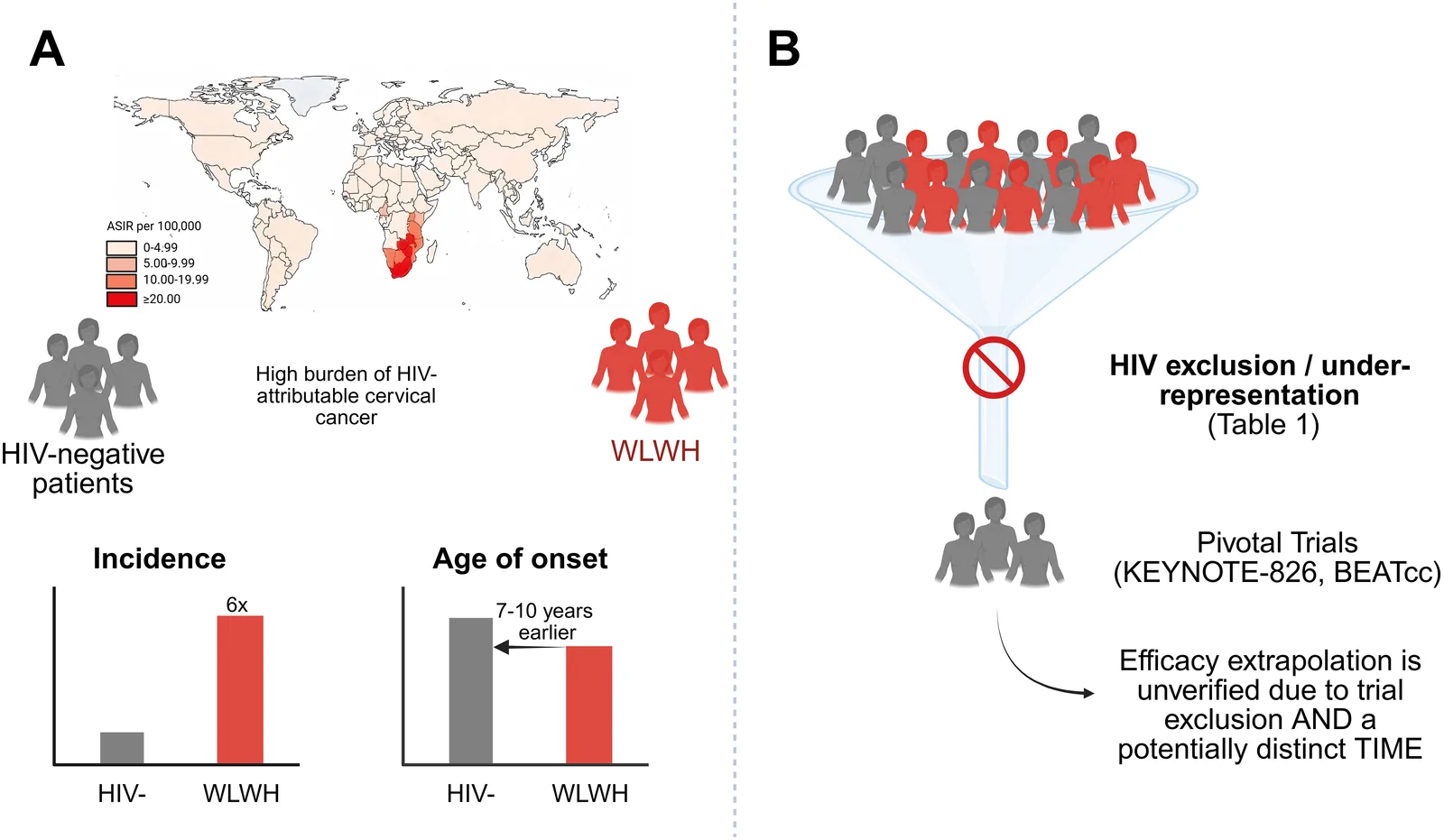
The epidemiological polarization and clinical extrapolation dilemma. **(A)** Women living with HIV (WLWH) face a disproportionate global burden. The incorporated global map, illustrating the country-level age-standardized incidence of HIV-attributable cervical cancer, is reproduced from Stelzle et al. (10). Epidemiologically, WLWH exhibit an approximately 6-fold higher incidence (9, 10) and experience an onset of invasive disease 7–10 years earlier (11, 16) compared to HIV-negative populations. **(B)** The resulting clinical extrapolation dilemma. Despite the high burden, WLWH are systemically excluded from or underrepresented in pivotal immunotherapy trials (e.g., KEYNOTE-826, BEATcc). Consequently, extrapolating efficacy data to this population remains unverified, driven both by insufficient trial representation and the potentially distinct immunobiology of the HIV-associated tumor immune microenvironment.

The widespread scale-up of antiretroviral therapy (ART) has transformed HIV into a chronic, manageable condition, prolonged life expectancy, and reduced—but not eliminated—the risk of invasive cervical cancer (17, 18). Critically, the immunological benefits of ART are compartmentalized. While peripheral CD4^+^ T-cell counts may recover and HIV viral load may become durably suppressed, mucosal immune surveillance in the cervicovaginal tract remains persistently impaired (19). Tissue-resident memory T cells fail to fully reconstitute, dendritic-cell cross-presentation is durably compromised, and cervicovaginal microbial dysbiosis fuels chronic local inflammation (20, 21). The downstream consequence is a uniquely permissive niche in which hrHPV persists across multiple genotypes, progresses more rapidly through pre-invasive lesions, and ultimately establishes invasive disease within an immunologically reprogrammed substrate (22).

These observations converge on a central thesis: the tumor immune microenvironment (TIME) of HIV-associated cervical cancer is not merely a quantitatively depleted version of its HIV-negative counterpart, but hypothesized to be a potentially distinct immunological landscape. It is shaped by compounded T-cell exhaustion driven by chronic dual antigen exposure (23), paracrine signaling from HIV accessory proteins to HPV-transformed epithelium (24), defective antigen presentation arising from both viral programs (25, 26), and a tolerogenic myeloid- and microbiome-skewed stroma (27). Each of these dimensions has direct implications for ICI responsiveness, biomarker selection, and the design of combination strategies—yet none has been systematically incorporated into current clinical paradigms.

We therefore propose that HIV-associated cervical cancer be investigated as a potentially distinct and clinically heterogeneous immunological context, requiring dedicated mechanistic, translational, and clinical study rather than uncritical extrapolation from predominantly HIV-negative cohorts.In this Review, we (1) delineate the epidemiological and clinical features of HIV-associated cervical cancer in the ART era (Section 2); (2) examine the host-mediated biological interactions linking HIV-associated immune dysfunction to HPV persistence and cervical carcinogenesis (Section 3); (3) propose a four-axis reference framework for the HPV-driven cervical tumor immune microenvironment (TIME) (Section 4) and map potential HIV-associated perturbations onto this framework to derive a provisional five-domain IEM phenotype in WLWH (Section 5); (4) critically appraise the available evidence for immune checkpoint inhibitor (ICI) therapy and outline hypothesis-driven therapeutic and trial-design strategies (Section 6); (5) propose an exploratory multidimensional biomarker framework for characterizing the IEM phenotype (Section 7); (6) introduce a provisional, risk-adapted, and longitudinal research-stratification framework for future studies (Section 8); and (7) outline a question-driven portfolio of next-generation preclinical models for mechanistic validation and translational development (Section 9).

## 2 Literature search strategy

To support this narrative, mechanism-oriented Review, we searched PubMed/MEDLINE and Web of Science for English-language articles published from database inception to May 1, 2026. Search terms combined MeSH terms, where applicable, with free-text keywords across three conceptual domains: (i) population—”HIV,” “human immunodeficiency virus,” “PLWH,” “people living with HIV,” “women living with HIV,” “WLWH,” and “AIDS”; (ii) disease—“cervical cancer,” “cervical neoplasm,” “cervical intraepithelial neoplasia,” “HPV,” and “human papillomavirus”; and (iii) intervention or mechanism—“immunotherapy,” “immune checkpoint inhibitor,” “PD-1,” “PD-L1,” “tumor immune microenvironment,” “mucosal immunity,” “tissue-resident memory T cell,” and “cervicovaginal microbiome.” ClinicalTrials.gov was additionally searched using combinations of terms related to HIV, cervical cancer, and immune checkpoint inhibition.

We prioritized pivotal phase II/III clinical trials, prospective clinical cohorts, translational and mechanistic studies, and recent systematic reviews or meta-analyses. Case reports, conference abstracts without subsequent peer-reviewed publication, and non-English-language articles were not considered. HIV-related eligibility criteria for selected phase II/III cervical cancer immunotherapy trials were manually verified using ClinicalTrials.gov records, published protocols, and supplementary appendices, where available. Reference lists of relevant articles and reports from the World Health Organization, UNAIDS, and the International Agency for Research on Cancer were also screened to identify additional sources.

This article was designed as a narrative, mechanism-oriented Review rather than a systematic review. Accordingly, study selection was not conducted according to PRISMA, and no formal risk-of-bias assessment or meta-analysis was performed.

## 3 Epidemiological and clinical landscape: a persistent burden despite ART

The global rollout of antiretroviral therapy (ART) has fundamentally altered the trajectory of HIV, yet it has not dissolved the epidemiological synergy between HIV and HPV that continues to fuel a disproportionate cervical cancer burden in women living with HIV (WLWH) (17, 18). Recent comprehensive population-based analyses (1990–2021) reaffirm the immense global burden and persistent geographic disparities of cervical cancer (28). Within this context, WLWH face an approximately six-fold higher incidence of cervical cancer than HIV-negative women, with more than 85% of attributable cases concentrated in sub-Saharan Africa (9, 12). This geographic clustering is not coincidental: it reflects the convergence of high HIV prevalence, limited screening coverage, and constrained access to both vaccination and oncologic care (9, 13). Cervical cancer thus remains an AIDS-defining malignancy whose elimination, as envisioned by the WHO 90-70–90 strategy (29), is biologically and operationally unattainable without HIV-tailored approaches.

The clinical phenotype of HIV-associated cervical cancer diverges from its HIV-negative counterpart along several mechanistically interconnected axes, as summarized in **Table 2**. Three features deserve particular emphasis.

**Table 2:** Reported differences between HIV-negative and HIV-associated cervical cancer: clinical, biological, and translational dimensions.

Dimension	HIV-negative	HIV-associated	Clinical/translational implications	Caveats and evidence quality	Refs
Global burden	~660, 000 cases/350, 000 deaths annually	~6-fold higher incidence; >85% of cases in sub-Saharan Africa	Cervical cancer elimination requires HIV-tailored strategies	Estimates heterogeneous across regions; influenced by screening coverage	(9, 10, 12, 28)
Age at diagnosis	Median 50–55 y	Median 40–45 y; up to 7–10 y earlier	Earlier screening initiation	Age differences confounded by population demographics and life expectancy	(10, 16)
HPV profile	HPV16/18 ~70%; single-genotype	Multi-genotype, non-16/18 enriched	Genotype-inclusive vaccination and screening	Genotype distribution varies by geography; small cohorts limit inference	(19, 29, 30)
HPV persistence and clearance	24-mo clearance ~60–70%	24-mo clearance ~30–40%	HPV-based primary testing	Definitions of clearance vary; influenced by ART status	(11, 29, 31)
CIN progression	Lower progression to CIN2/3	2- to 4-fold higher; CD4-dependent	Lower colposcopy threshold	Most data from observational cohorts; treatment access bias	(31–33)
Effect of ART	NA	Reduces HSIL progression ~40%; does not normalize risk	ART integration with screening	Residual risk depends on ART duration, adherence, and timing	(17, 18, 33)
Mucosal immunity	Intact compartmentalized immunity	Persistent CD4^+^/CD8^+^ TRM depletion; chronic local inflammation	Compartment-specific biomarkers needed	Most mechanistic data from peripheral blood, not tumor tissue	(20, 34–36)
TIME composition	Variable PD-L1; inflamed/excluded phenotypes	Compounded exhaustion; myeloid skewing; defective antigen presentation	Distinct biomarker frameworks required	Direct comparative single-cell/spatial data in invasive WLWH tumors lacking	(20, 23, 37)
Cervicovaginal microbiome	L. crispatus–dominant CST I	CST IV dysbiosis; pro-inflammatory milieu	Microbiome-targeted adjuncts	Causality vs association unresolved	(20, 21, 38, 39)
Treatment outcomes	Stage- and biology-determined	Additionally shaped by CD4, ART, and structural barriers; 2-y OS ~65% vs ~79%	Integrated HIV–oncology pathways	Outcome gaps partly attributable to care disparities, not biology alone	(10, 13, 40–42)
Immunotherapy evidence	Phase III–supported (KEYNOTE-826, EMPOWER-Cervical 1, BEATcc, KEYNOTE-A18)	Exclusion or underrepresentation in pivotal trials	HIV-inclusive trial design with pre-specified subgroups	Limited safety data from CITN-12 and similar small studies	(1–4, 14, 15)

Numerical estimates are approximate ranges synthesized from heterogeneous studies and do not represent direct head-to-head comparisons. The reported differences may be influenced by geography, screening coverage, ART access, disease stage, and structural disparities in cancer care.

ART, antiretroviral therapy; CD4, cluster of differentiation 4; CD8, cluster of differentiation 8; CIN, cervical intraepithelial neoplasia; CST, community state type; HIV, human immunodeficiency virus; HPV, human papillomavirus; HSIL, high-grade squamous intraepithelial lesion; mo, months; NA, not applicable; OS, overall survival; PD-L1, programmed death-ligand 1; TIME, tumor immune microenvironment; TRM, tissue-resident memory; WLWH, women living with HIV; y, years.

First, HIV reshapes the natural history of HPV infection itself: WLWH exhibit reduced spontaneous clearance of high-risk HPV (hrHPV) (approximately 30–40% at 24 months vs approximately 60–70% in HIV-negative women) (11, 30), an expanded genotype repertoire enriched for non-16/18 types such as HPV35, 52 and 58 (19, 31), and a markedly higher rate of persistent multi-genotype coinfection (43). These shifts have direct preventive implications, providing the rationale for nonavalent vaccination, HPV-based primary screening, and genotype-inclusive surveillance algorithms (32, 44).

Second, persistent virological insult under conditions of mucosal immunoparesis accelerates the virological-to-neoplastic transition: progression from cervical intraepithelial neoplasia grade 1 (CIN1) to CIN2/3 occurs two- to four-fold more frequently in WLWH and is steeply dependent on CD4 nadir and HIV viral load (16, 40). Consequently, invasive disease in WLWH presents at a median age of 40–45 years—approximately seven to ten years earlier than in HIV-negative women (10, 34)—underscoring the need for earlier screening initiation and lower colposcopic referral thresholds (**Figure 1A**).

Third, although ART substantially reduces the incidence of invasive cervical cancer and lowers the risk of high-grade squamous intraepithelial lesion (HSIL) progression by approximately 40%, it does not normalize risk to that of HIV-negative women, even after sustained virological suppression (16–18). The biological basis for this residual risk lies in compartmentalized immune reconstitution: while peripheral CD4^+^ T-cell counts may recover, cervicovaginal tissue-resident memory T cells, dendritic-cell function, and mucosal cytokine homeostasis remain durably perturbed (20, 35, 36, 45). This “mucosal immunological blind spot” sustains a permissive niche for HPV persistence and progression, and—as we develop in subsequent sections—reprograms the tumor immune microenvironment in ways that have direct implications for immunotherapeutic response (20, 23, 41).

Clinical outcomes amplify these biological asymmetries. In the Botswana cohort, two-year overall survival among WLWH with locally advanced cervical cancer treated with chemoradiation was approximately 65%, compared with 79% in HIV-negative women (33, 42). While part of this gap reflects tumor-intrinsic biology and immune status, it is also driven by structural determinants of care—screening access, radiotherapy availability, ART continuity, and socioeconomic barriers (10, 13, 46). Disentangling biological from structural contributors is essential, because it determines whether interventions should target tumor biology, healthcare systems, or both.

Taken together, these epidemiological, virological, and clinical divergences argue against treating HIV-associated cervical cancer as a simple variant of conventional disease. Taken together, these epidemiological, virological, and clinical observations argue against assuming complete biological equivalence between HIV-associated and HIV-negative cervical cancer. They provide a rationale for HIV-tailored screening and follow-up strategies (32, 44), as well as for dedicated investigation of whether residual systemic and mucosal immune perturbations modify tumor immunity, biomarker performance, or treatment response. The host-mediated mechanisms potentially linking HIV infection to HPV persistence and cervical carcinogenesis are examined in Section 3.

## 4 Host-mediated HIV–HPV interactions in cervical carcinogenesis

The compartmentalized mucosal immune dysfunction described in Section 2 sets the stage for a fundamentally distinct biological question: how does HIV—a virus that does not productively infect cervical epithelium—nonetheless reshape HPV-driven carcinogenesis at the molecular level? The progression from HPV acquisition to invasive cervical carcinoma in women living with HIV (WLWH) cannot be reduced to the additive consequence of two independent viral life cycles. It is, instead, the emergent outcome of a paracrine virological interactome in which HIV-infected immune cells and HPV-infected epithelial cells, despite their distinct cellular tropisms, engage in sustained cross-compartmental molecular dialogue (22, 24). This dialogue establishes an immunologically permissive niche for HPV persistence and oncogenic progression, operating across three mechanistically interconnected axes. Because the molecular evidence underpinning each axis varies considerably in robustness, we explicitly stratify the supporting data across four evidence tiers (**Table 3**) to delineate where mechanistic certainty ends and translational hypothesis begins.

**Table 3:** Evidence stratification for the HIV–HPV oncogenic interactome: from clinical observation to molecular hypothesis.

Evidence tier	Specific claim	Strength of supporting data	Key references
Tier 1 — Established clinical and epidemiological evidence	HIV is associated with reduced spontaneous HPV clearance at 24 months, persistent infection with multiple HPV genotypes, and a two- to fourfold higher rate of progression from CIN1 to CIN2–3.	Large prospective cohorts and meta-analyses	(11, 29, 31–33)
Tier 2 — Supportive human translational evidence	ART reduces but does not normalize cervical cancer risk. Persistent mucosal immune dysfunction, impaired dendritic-cell function, and cervicovaginal microbiome dysbiosis provide plausible biological explanations for this residual risk.	Translational cohorts, ex vivo immunological studies, and longitudinal microbiome studies	(17, 18, 20, 21, 34, 35)
Tier 3 — Preclinical mechanistic evidence	HIV accessory proteins may influence HPV oncogene transcription and epithelial cell-cycle control through paracrine or EV-mediated mechanisms. Nef-mediated modulation of antigen presentation is established primarily in HIV-infected immune cells.	*In vitro* keratinocyte and coculture models, with limited ex vivo support	(22, 24, 46–49)
Tier 4 — Hypothesis-generating and unresolved	Direct spatial crosstalk between HIV-infected stromal cells and HPV-transformed epithelium in invasive human cervical tumors, and metabolic coupling between tumor-derived lactate and local HIV reservoir activity, remain unvalidated.	Indirect biological plausibility and limited correlative evidence; awaits single-cell and spatial multi-omic validation	(50–55)

Evidence tiers reflect the type and maturity of supporting evidence within this narrative framework and should not be interpreted as a formal certainty-of-evidence grading system. References listed for Tier 4 provide indirect biological or mechanistic support rather than direct validation of the proposed interactions in intact human tumors.

ART, antiretroviral therapy; CIN, cervical intraepithelial neoplasia; EV, extracellular vesicle; HIV, human immunodeficiency virus; HPV, human papillomavirus; mo, months.

To clarify the translational readiness of our mechanistic claims, we employ a manuscript-specific evidence-labeling scheme (Evidence Tier E1–E4): E1 (Direct evidence from human HIV-associated cervical cancer specimens or cohorts); E2 (Human evidence from HIV-negative cervical cancer, HIV infection, or other HPV-associated cancers); E3 (Preclinical evidence from *in vitro*, ex vivo, or animal models); E4 (Mechanistic hypothesis or expert inference without direct supporting data).

### 4.1 Axis 1 | paracrine transactivation via extracellular vesicle networks

The canonical oncogenic engine of HPV—E6-mediated p53 degradation and E7-mediated pRb inactivation—operates independently of HIV status (Evidence Tier: E1, established human biology) (25, 26). However, accumulating preclinical evidence suggests that HIV-1 accessory proteins, most notably the transactivator of transcription (Tat), can be released from infected stromal immune cells and internalized by adjacent epithelial cells through extracellular vesicle (EV)–mediated transfer or direct uptake (Evidence Tier: E3, *in vitro* models) (47, 48). Once internalized, Tat has been shown to bind the HPV-16 long control region (LCR) and to increase E6/E7 promoter activity in *in vitro* preclinical keratinocyte models (Evidence Tier: E3, *in vitro* data) (22, 49). Parallel evidence implicates HIV Vpr in modulating epithelial cell-cycle checkpoints and Nef in altering host cell signalling cascades that converge on NF-κB and STAT3 activation (Evidence Tier: E3, preclinical evidence) (24, 50). Although these mechanisms are biologically plausible, their quantitative contribution within invasive human cervical tumors remains unknown and should be regarded as provisional (Evidence Tier: E4).

### 4.2 Axis 2 | metabolic and microenvironmental coupling

HPV E6/E7 oncoproteins induce a pronounced Warburg phenotype in cervical epithelium, characterized by enhanced aerobic glycolysis, lactate efflux, and acidification of the surrounding stroma (Evidence Tier: E2, human translational studies) (51, 52). The resulting lactate-rich, acidic microenvironment exerts well-documented suppressive effects on cytotoxic T-lymphocyte function, dendritic cell maturation, and myeloid cell polarization (Evidence Tier: E2, ex vivo human models) (53, 54). In the HIV-coinfected setting, this metabolic landscape may be further amplified by chronic immune activation and ART-associated mitochondrial perturbations, although direct evidence linking tumor-derived lactate to enhanced local HIV reservoir activity in cervical tissue remains limited and hypothesis-generating (Evidence Tier: E4, provisional hypothesis) (56). What is more firmly established is that HPV-driven metabolic reprogramming and HIV-associated mucosal inflammation can each promote an immunosuppressive, pro-angiogenic microenvironment resistant to cytotoxic immune clearance (Evidence Tier: E2, human ex vivo data) (57, 58).

### 4.3 Axis 3 | convergent impairment of antigen presentation

Effective immune control of HPV-infected and transformed cells depends critically on intact HLA class I antigen presentation and dendritic cell cross-presentation (Evidence Tier: E1) (59). HPV E5 and E7 oncoproteins directly impair HLA class I trafficking through downregulation of TAP1, ERAP1, and the heavy chain itself, reducing the antigen-presentation machinery in *in vitro* epithelial models (Evidence Tier: E3) (55, 60). In WLWH, this epithelium-intrinsic evasion is compounded by HIV-driven defects in the stromal antigen-presenting compartment: depletion of CD4+ helper T cells impairs CD8+ T-cell priming, and altered cytokine gradients (notably elevated IL-10 and TGF-β, reduced IL-12) skew responses toward tolerance (Evidence Tier: E2) (61–63). Extrapolating from the established general role of cDC1 cells in cross-presentation, and acknowledging the lack of direct evidence in intact HIV-associated cervical tumors, we hypothesize that conventional dendritic cell cross-presentation capacity may be further compromised (Evidence Tier: E4). Whether extracellular or EV-associated Nef directly alters antigen-presenting cells in the cervical stroma remains uncertain (Evidence Tier: E4) (64).

Taken together, these three axes describe a molecular framework in which HIV does not merely permit HPV oncogenesis through systemic immunosuppression, but actively co-conspires with HPV through paracrine signalling, metabolic coupling, and convergent immune evasion (**Figure 2**). However, the evidence base is heterogeneous: the individual HPV- and HIV-associated mechanisms are supported to varying degrees by clinical, translational, and preclinical studies, whereas their integrated operation within intact HIV-associated cervical tumors remains poorly resolved. Bridging this gap is, in our view, the single most important translational priority for the field, and we return to specific experimental approaches in Section 7.

**Figure 2:**
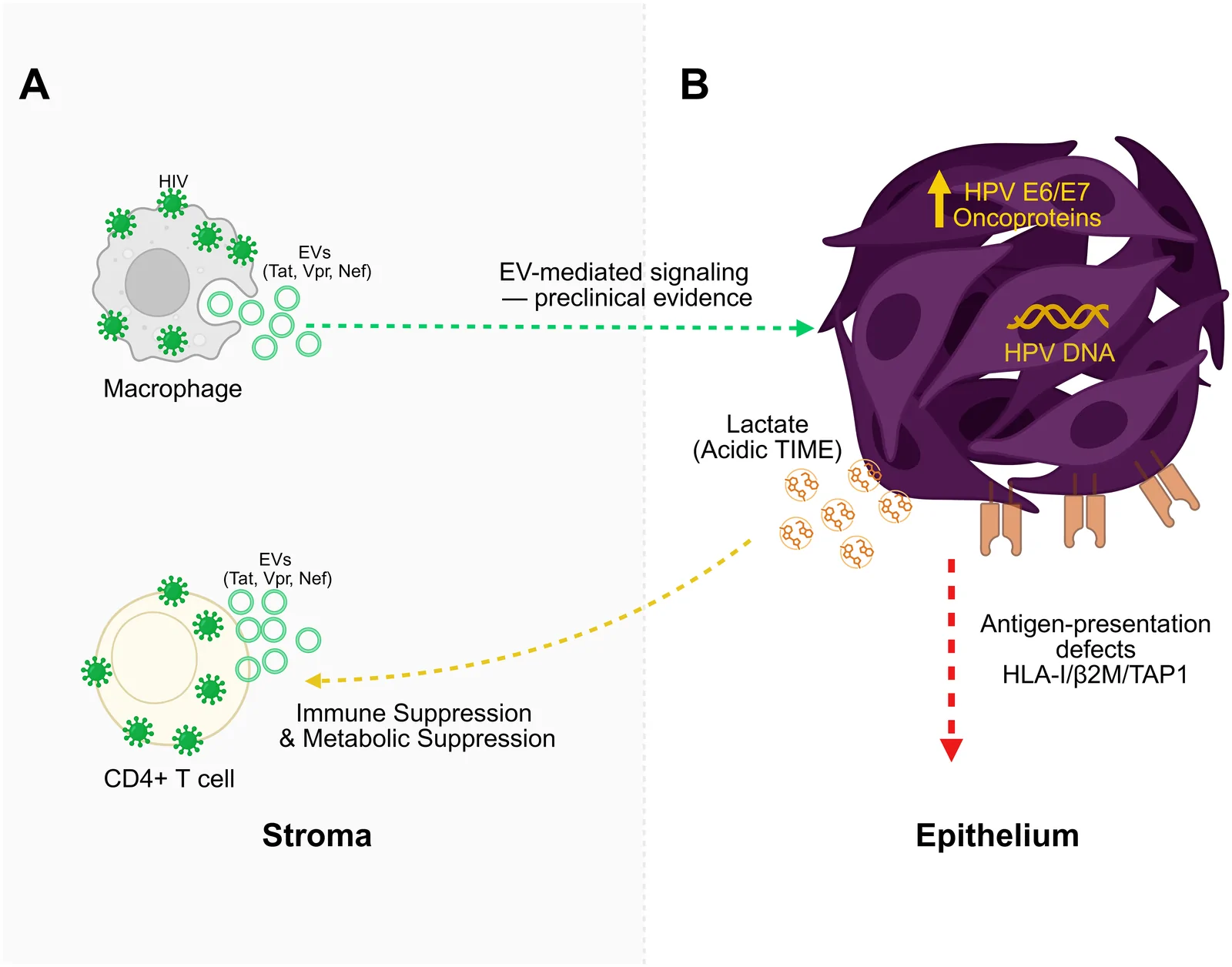
Proposed cross-spatial molecular crosstalk between HIV-infected stroma and HPV-driven epithelium. **(A)** Stromal Compartment: HIV-infected macrophages and CD4+ T cells release extracellular vesicles (EVs) containing viral accessory proteins (Tat, Vpr, Nef). **(B)** Epithelial Compartment: These EVs are hypothesized to penetrate the epithelial layer. Based on *in vitro* and preclinical evidence (denoted by dashed arrows), this cross-compartmental signaling may accelerate HPV E6/E7 oncoprotein transcription and further exacerbate HPV-mediated antigen-presentation defects (e.g., HLA-I/B2M/TAP1 downregulation). Conversely, the HPV-driven tumor exerts metabolic suppression on local immune cells via excessive lactate accumulation, creating a profoundly acidic and immunosuppressive microenvironment.

## 5 The cervical cancer tumor immune microenvironment: a four-axis framework as the immunological substrate for therapy

The host-mediated interactions described in Section 3 ultimately converge within a structured, multicellular ecosystem—the cervical cancer tumor immune microenvironment (TIME). Before examining how HIV may perturb this ecosystem in Section 5, it is useful to establish a reference framework for the HPV-driven cervical TIME against which potential HIV-associated alterations can be evaluated. We deconstruct the cervical TIME along four functionally distinct but mechanistically interdependent axes, progressing from cellular effector–suppressor balance and molecular checkpoint regulation to the mucosal and microbial foundations of local immunity (**Figure 3**). For each axis, we highlight features of particular relevance to WLWH that are developed further in Section 5.

**Figure 3:**
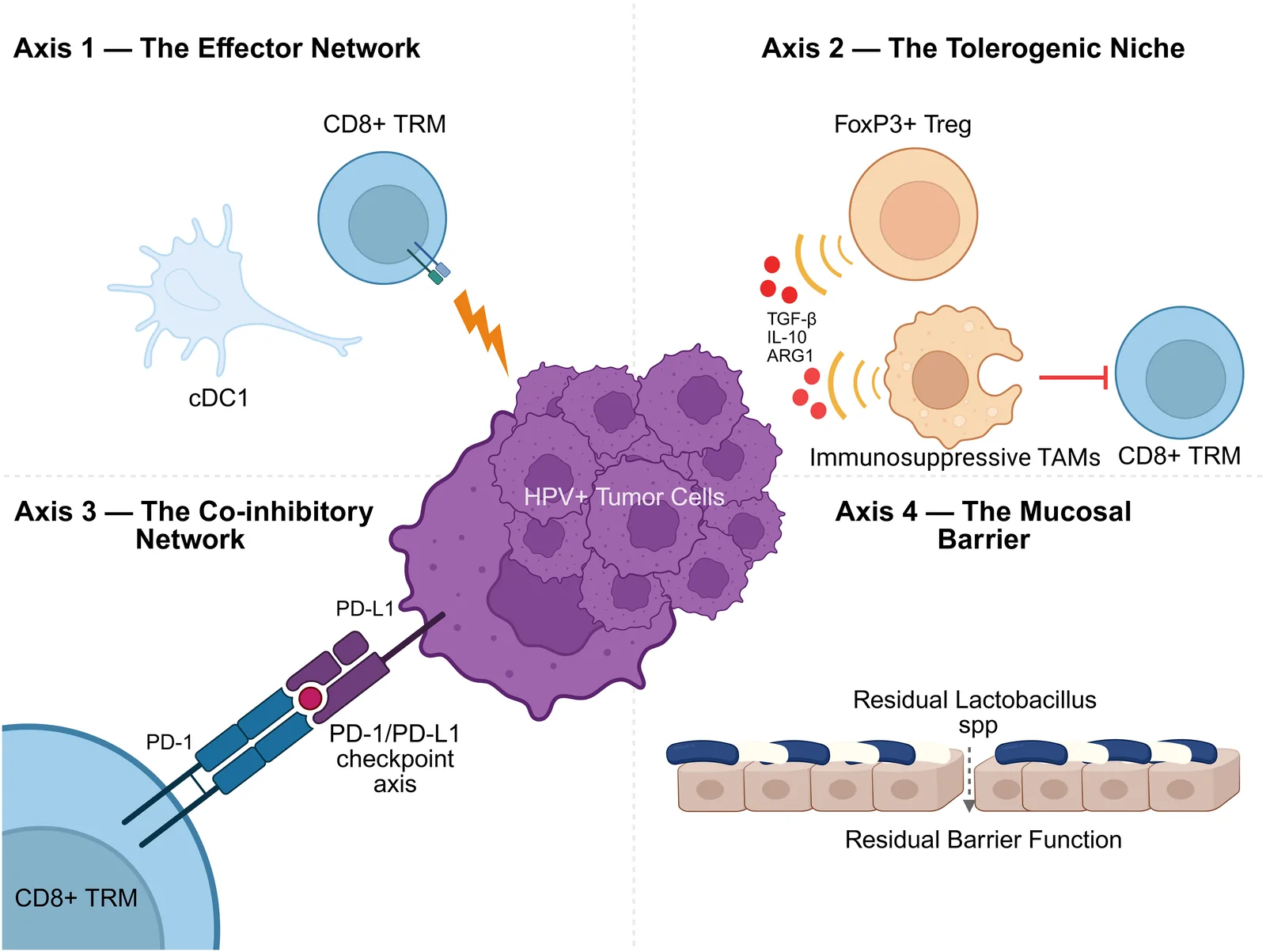
The four-axis baseline architecture of the HPV-driven cervical tumor immune microenvironment (TIME). It is crucial to emphasize that this “baseline” represents the immunological state of established, invasive cervical cancer, rather than a healthy cervicovaginal mucosa. Consequently, a baseline degree of mucosal disruption and immune evasion is inherently present even prior to HIV infection. In the absence of HIV-induced disruption, this baseline TIME is governed by four interwoven axes surrounding the HPV+ tumor cells: The Effector Network (Axis 1), driven by CD8+ tissue-resident memory T cells (TRM) and cDC1s; The Tolerogenic Niche (Axis 2), dominated by FoxP3+ regulatory T cells (Tregs) and immunosuppressive tumor-associated macrophages (TAMs) secreting TGF-β, IL-10, and ARG1; The Co-inhibitory Network (Axis 3), characterized by the PD-1/PD-L1 checkpoint regulation; and The Mucosal Barrier (Axis 4), defined by residual barrier function and residual Lactobacillus spp., reflecting the compromised mucosal state of invasive disease.

### 5.1 Axis 1 — the effector and antigen-presenting network

Immune control of HPV-driven transformation depends on conventional type 1 dendritic cells (cDC1s) cross-presenting HPV-derived antigens to CD8^+^ cytotoxic T lymphocytes (65). Within the tumor, a subset of activated CD8^+^ T cells acquires a CD103^+^CD69^+^ tissue-resident memory T-cell phenotype. Higher densities of these cells have been associated with favourable survival outcomes in cervical cancer, although their predictive value for ICI benefit remains incompletely established (66, 67). NK cells provide a complementary, antigen-presentation-independent cytotoxic pathway, particularly against cells with reduced HLA class I expression (68). As discussed in Section 5, evidence from HIV immunology suggests that this network may be compromised by persistent dendritic-cell dysfunction, enhanced CD8^+^ T-cell exhaustion, and impaired NK-cell activity (27).

### 5.2 Axis 2 — the tolerogenic and regulatory niche

Counterbalancing the effector network is an immunoregulatory compartment comprising FOXP3^+^ Tregs, MDSCs, and immunosuppressive TAM states (69). These populations suppress antitumor immunity through TGF-β and IL-10 secretion, ARG1-mediated arginine depletion, IDO1-mediated tryptophan catabolism, and, in some tumors, CXCL12–CXCR4-associated immune exclusion (70, 71). A high intratumoral Treg-to-CD8 (+) T-cell ratio has been associated with adverse clinical outcomes in HPV-associated head and neck squamous cell carcinoma (72), suggesting a potentially similar prognostic relevance in cervical cancer. As explored in Section 5, relative enrichment of regulatory T-cell and suppressive myeloid programs may persist in WLWH despite systemic CD4^+^ T-cell depletion (23).

### 5.3 Axis 3 — the co-inhibitory network and T-cell exhaustion

Persistent viral and tumor-antigen exposure can promote a spectrum of T-cell dysfunction characterized by co-expression of inhibitory receptors—including PD-1, CTLA-4, TIM-3, LAG-3, and TIGIT—and progressive loss of effector function (23, 38). PD-L1 may also be expressed by both tumor and myeloid cells (69). In persistent, recurrent, or metastatic cervical cancer, a PD-L1 CPS ≥1 identifies patients eligible for first-line pembrolizumab combined with chemotherapy, with or without bevacizumab, in relevant regulatory settings (1). However, CPS has limited discriminatory precision and does not fully distinguish responsive from resistant tumors. This limitation may be particularly relevant in WLWH, in whom HIV-associated inflammation could induce PD-L1 expression independently of productive antitumor immunity; this possibility remains to be validated in HIV-associated cervical tumors (19, 38).

### 5.4 Axis 4 — the mucosal barrier and cervicovaginal microbial ecology

The cervicovaginal microbiome is an important ecological determinant of the mucosal environment in which the cervical TIME develops (39). Lactobacillus crispatus–dominant communities, commonly classified as CST I, are generally associated with lower mucosal inflammation and greater ecological stability (73). By contrast, diverse anaerobe-enriched communities, often classified as CST IV, have been associated with increased inflammatory cytokines, enrichment of activated CCR5^+^ CD4^+^ T cells, reduced HPV clearance, and a higher risk of cervical lesion persistence or progression (74, 75).

Dysbiosis-associated microbial products and enzymes may disrupt epithelial tight junctions, increase mucosal permeability, and enhance local exposure to pro-inflammatory microbial ligands (76). Sustained pattern-recognition receptor signalling, including TLR4/NF-κB activation, may promote cytokine and chemokine programs that favour the recruitment or polarization of suppressive myeloid populations, thereby linking microbial dysbiosis to Axis 2 (20). In WLWH, CST IV communities appear more prevalent and may persist despite ART, potentially contributing to an inflamed but dysfunctional mucosal phenotype (77, 78). Direct evidence linking these community states to ICI outcomes in HIV-associated cervical cancer remains limited.

Taken together, these four axes constitute a layered architecture in which checkpoint networks and microbial–mucosal ecology shape the balance between cellular effectors and suppressors. HIV may remodel all four dimensions through coordinated and mutually reinforcing processes centred on persistent mucosal immune dysfunction, thereby generating a TIME state that is qualitatively distinct from that of HIV-negative cervical cancer. This HIV-associated immunological signature is examined in Section 5.

## 6 HIV-associated remodeling of the cervical cancer immune ecosystem: from five perturbation domains to the proposed IEM phenotype

Building on the four-axis baseline framework established in Section 4, we next consider how HIV may remodel the cervical cancer TIME and whether this remodeling generates a biologically distinct and clinically relevant immune state. We address this question in two steps. First, we propose five overlapping perturbation domains that map onto the baseline TIME architecture, including a cross-cutting domain representing chronic inflammatory processes characteristic of treated HIV infection. Second, we integrate these domains into a testable conceptual archetype—the inflamed, exhausted, and mucosally disrupted (IEM) phenotype—which may not be adequately captured by conventional “hot” and “cold” tumor classifications.

### 6.1 The five-domain perturbation model

The proposed mapping between the four baseline axes and five HIV-associated perturbation domains is summarized in **Table 4**. These domains are overlapping processes rather than mutually exclusive categories. Domains 1–4 map primarily, but not exclusively, onto components of the baseline TIME architecture, whereas Domain 5 represents a cross-cutting inflammatory state capable of modifying all four axes.

**Table 4:** Mapping the proposed five-domain HIV perturbation model onto the baseline cervical tumor immune microenvironment.

Baseline TIME architecture	Proposed HIV-associated domain	Representative alteration	Candidate research readout	Evidence status in HIV-associated cervical cancer
Effector and co-inhibitory networks	Domain 1: compounded T-cell exhaustion	Shift toward differentiated or terminally exhausted CD8^+^ T-cell states and reduced polyfunctionality	Multiplex spatial assessment of PD-1, TIM-3, LAG-3, and TIGIT co-expression; ratio of TOX-high/TCF1-low to TCF1^+^ progenitor-exhausted CD8^+^ T cells	Limited direct evidence; supported mainly by broader HIV and cancer exhaustion studies
Effector and APC compartments	Domain 2: antigen-presentation impairment	cDC1 dysfunction and reduced or heterogeneous tumor-cell HLA-I/B2M/TAP1 expression	cDC1 density and spatial distribution; cross-presentation signatures; tumor-cell HLA-I/B2M/TAP1 expression	Predominantly indirect evidence from HIV immunology and HPV-associated cervical cancer
Tolerogenic niche	Domain 3: myeloid-regulatory skewing	Enrichment of immunosuppressive TAM states, Tregs, and suppressive metabolic pathways	Spatial TAM/Treg/CD8^+^ profiles; ARG1 and IDO1 expression; candidate kynurenine and adenosine-pathway measures	Supportive general cervical cancer evidence; limited evidence for HIV-specific augmentation
Mucosal barrier and microbial ecology	Domain 4: barrier disruption and dysbiosis	Lactobacillus depletion, anaerobe-enriched community states, and impaired epithelial-barrier integrity	Cervicovaginal microbiome profile; epithelial tight-junction integrity; local inflammatory markers	Supportive HIV and cervicovaginal mucosal evidence; limited tumor-specific validation
Cross-cutting domain across all axes	Domain 5: chronic bystander inflammation	Persistent type I/II IFN-response programs, microbial translocation, and systemic myeloid activation	Intratumoral IFN-response and myeloid-activation signatures; plasma sCD14 and LBP as complementary systemic measures	Strong evidence in chronic HIV infection, but limited validation within the cervical TIME

The five domains are overlapping components of a proposed research framework rather than mutually exclusive biological categories. Evidence status refers specifically to validation in HIV-associated cervical cancer; several proposed alterations are extrapolated from broader studies of chronic HIV infection, HPV-associated cervical cancer, or mucosal immunology. The listed readouts have not been validated as clinical biomarkers or treatment-selection criteria.

APC, antigen-presenting cell; ARG1, arginase 1; β2M, beta-2-microglobulin; cDC1, conventional type 1 dendritic cell; CST, community state type; HIV, human immunodeficiency virus; HLA-I, human leukocyte antigen class I; IDO1, indoleamine 2,3-dioxygenase 1; IFN, interferon; LAG-3, lymphocyte-activation gene 3; LBP, lipopolysaccharide-binding protein; PD-1, programmed cell death protein 1; sCD14, soluble CD14; TAM, tumor-associated macrophage; TAP1, transporter associated with antigen processing 1; TCF1, T-cell factor 1; TIGIT, T-cell immunoreceptor with immunoglobulin and ITIM domains; TIME, tumor immune microenvironment; TIM-3, T-cell immunoglobulin and mucin-domain containing 3; TOX, thymocyte selection-associated high-mobility group box; Treg, regulatory T cell.

Domain 1—Compounded T-cell exhaustion

CD8^+^ T-cell dysfunction in HIV-associated cervical cancer may reflect the interaction of several partially overlapping drivers: persistent HIV-associated immune activation during suppressive ART, continued exposure to HPV-derived antigens, tumor-derived suppressive signals, and incomplete restoration of antigen-specific T-cell repertoire and function (35, 37, 59, 62, 71).

Single-cell studies of chronic viral infection and cancer have identified an exhaustion continuum that includes TCF1^+^ progenitor-exhausted populations with renewal and proliferative capacity and more differentiated TOX-high, TCF1-low populations with limited reinvigoration after PD-1 blockade (79, 80). Extrapolating from these models and emerging mucosal datasets in WLWH (23), we hypothesize that HIV-associated cervical tumors may exhibit a shift toward more differentiated exhaustion states, potentially involving HPV-specific CTLs. If confirmed, such a shift could help explain attenuated responses to PD-1 monotherapy and would support evaluating the progenitor-exhausted pool as a candidate determinant of ICI responsiveness.

#### 6.1.1 Domain 2—two-layered antigen-presentation impairment

Antigen-presentation impairment may operate through two mechanistically distinct but convergent layers. Because productive HIV infection of cervical epithelial tumor cells has not been convincingly demonstrated as a major feature of the disease, HIV-associated effects are likely to occur primarily through immune and stromal compartments.

The first layer is HPV-driven immune evasion. HPV E5 can interfere with HLA class I trafficking, whereas E7 can suppress components of the antigen-processing machinery, including TAP1, contributing to reduced or heterogeneous HLA class I expression (60, 68). The second layer is indirect HIV-associated impairment. Evidence from HIV immunology suggests altered dendritic-cell abundance or function, incomplete restoration during ART, and HIV protein-associated disruption of antigen processing or HLA class I trafficking (61, 64, 81). Dysregulated CXCL9/10 gradients may further impair the recruitment or spatial colocalization of dendritic cells and T cells (82). Together, these processes could generate an HPV-antigen-bearing but inefficiently recognized tumor state.

#### 6.1.2 Domain 3—myeloid-regulatory skewing

HIV-associated immune dysfunction may favour the relative enrichment or reprogramming of FOXP3^+^ Tregs, immunosuppressive TAM states, and MDSCs (23, 83). Extrapolating from findings in HPV-associated head and neck squamous cell carcinoma (72) and broader solid tumor immunology (84), we hypothesize that similar suppressive pathways involving IDO1, ARG1, and CD39/CD73 may be enriched in the cervical TIME of WLWH. Whether these programs are quantitatively or spatially amplified in tumors from WLWH remains insufficiently defined. Apparent Treg enrichment despite systemic CD4^+^ T-cell depletion may reflect relative preservation of regulatory phenotypes within mucosal CD4^+^ compartments rather than an absolute increase in Treg numbers (85).

#### 6.1.3 Domain 4—mucosal barrier disruption and persistent dysbiosis

Experimental and mucosal studies suggest that HIV-associated proteins and chronic inflammation may impair epithelial tight-junction integrity, alter mucus properties, and promote anaerobe-enriched cervicovaginal communities that may persist despite suppressive ART (20, 76–78). These changes could increase mucosal permeability and local exposure to microbial products, activating pattern-recognition pathways that favour the recruitment or polarization of suppressive myeloid populations.

Clinical and interventional studies support an association between the gut microbiome and ICI outcomes in several cancers (86), but whether cervicovaginal dysbiosis modulates ICI response in cervical cancer remains unknown. By analogy, Lactobacillus-depleted communities could influence ICI responsiveness in WLWH, but this hypothesis requires prospective testing and should not currently guide treatment selection (87).

#### 6.1.4 Domain 5—chronic bystander inflammation

A characteristic feature of treated HIV infection is persistent, non-tumor-specific immune activation associated with microbial translocation, residual viral stimulation, coinfections, and age-associated inflammatory programs (35, 37). Within the cervical TIME, this state may have opposing effects. Acute or spatially restricted IFN-γ signalling can enhance antigen-processing machinery and PD-L1 expression, whereas persistent type I and/or type II IFN-response programs may promote adaptive resistance, myeloid activation, and immune dysfunction (88, 89). PD-L1 induction may therefore occur without an adequately functional tumor-specific T-cell response, creating what we term “immune visibility without immune efficacy.” Domain 5 is thus proposed as a cross-cutting state capable of reinforcing the other four domains.

### 6.2 Synthesizing the five domains: the proposed IEM phenotype and its operationalization

We propose that the integrated consequence of these five perturbation domains constitutes a biologically distinct and potentially clinically relevant immune-ecological state: the Inflamed, Exhausted, and Mucosally disrupted (IEM) phenotype (**Figure 4**). Crucially, the IEM acronym operates as a shorthand for the entire five-domain constellation. It does not conceptually omit antigen-presentation failure or myeloid-regulatory skewing; rather, these mechanisms are recognized as candidate biological mechanisms that may contribute to the ‘E’ (Exhaustion) and ‘I’ (Inflammation) components. Furthermore, the ‘M’ explicitly refers to the dominance of anaerobe-enriched (CST IV) cervicovaginal microbiomes that compromise epithelial barrier integrity.

**Figure 4:**
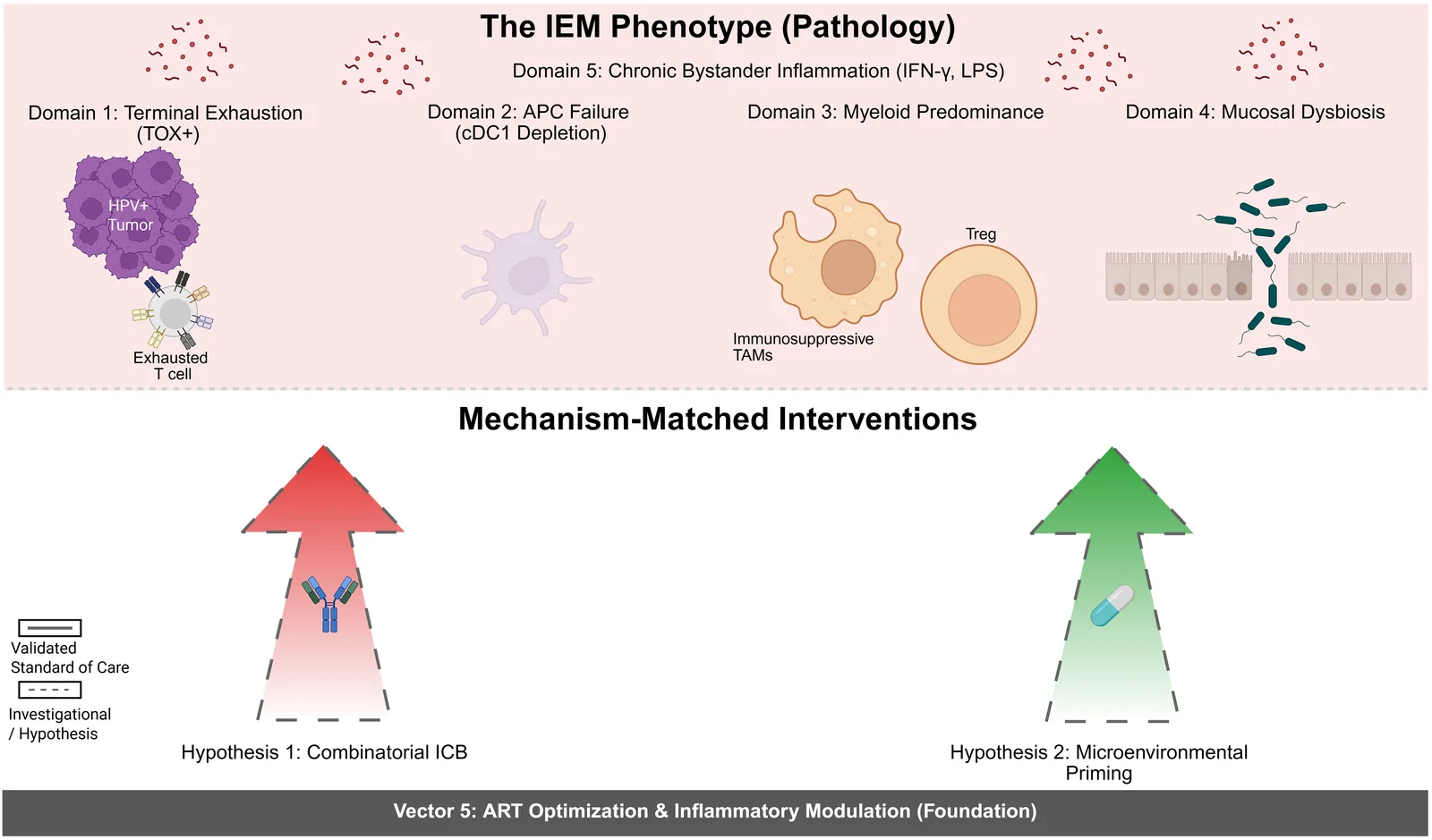
The proposed IEM phenotype and mechanism-matched combinatorial interventions. The upper panel delineates the conceptual domains of the IEM phenotype characterizing HIV-associated cervical cancer, including terminal T-cell exhaustion (Domain 1 & 2), myeloid predominance (Domain 3), mucosal dysbiosis (Domain 4), and chronic bystander inflammation (Domain 5). The lower panel maps the prioritized mechanism-matched therapeutic hypotheses designed to rewire the compromised TIME. To explicitly differentiate the translational readiness of these strategies, solid lines denote validated standard-of-care interventions (e.g., foundational ART optimization and standard single-agent immune checkpoint blockade for indicated patients). Conversely, dashed lines denote investigational or hypothesis-driven approaches (e.g., microbiome-directed therapies, dual-checkpoint blockade, and myeloid-regulatory interventions) that require rigorous prospective validation in WLWH.

Difference from the conventional “inflamed but exhausted” phenotype The proposed IEM framework is not intended to imply that inflammation or T-cell exhaustion is unique to cervical cancer in women living with HIV. Conventional “inflamed but exhausted” tumors are generally defined predominantly by local immune infiltration and dysfunctional T-cell states. In contrast, the IEM framework integrates these local features with systemic HIV-associated inflammation, mucosal ecological disruption, and candidate antigen-presentation and myeloid-regulatory mechanisms. Whether this integrated constellation is quantitatively enriched in WLWH or constitutes a biologically distinct phenotype remains unproven. We therefore present IEM as a testable multidimensional framework rather than an established HIV-specific disease entity.

We have revised the framework from a purely conceptual construct into a research-operationalizable model with candidate measurable components, while explicitly acknowledging that it is not yet a clinically validated classifier.

Operationalizing the IEM Phenotype: The IE-Core and M-Modifier The framework comprises an “IE” core with an “M” modifier. Thus, mucosal disruption is not required to recognize the core inflamed–exhausted state, but it is hypothesized to identify a biologically relevant IEM-M-positive subset.

The “I” Component: Provisionally defined by inflammatory activation accompanied by evidence of ineffective immune execution. The “I” component should not be inferred from an IFN-γ signature alone, because such signaling may also reflect productive antitumor immunity. It requires concurrent evidence of systemic inflammation. It is important to note that the Neutrophil-to-Lymphocyte Ratio (NLR) is not an IEM-specific biomarker, as it is influenced by active infections, OIs, corticosteroids, tumor burden, and ART; thus, it serves only as an auxiliary indicator (90).

The “E” Component: Operationalized through high-resolution cellular subpopulation refinement, assessing the ratio of terminally exhausted (TOX-high/TCF1-low) to progenitor-exhausted (TCF1-high) T cells within the TIME (91, 92).

The “M” Modifier: Operationalized via 16S rRNA sequencing (e.g., CST IV dominance) (20, 39) and markers of epithelial barrier permeability (e.g., sCD14) (35, 62).

Addressing Analytical Thresholds No universally validated numerical thresholds currently exist for these markers. Accordingly, IEM biomarkers should initially be analyzed as continuous variables. In exploratory cohorts, prespecified cohort-relative thresholds, such as the upper quartile or standardized z-scores, may be used for hypothesis testing, followed by independent threshold validation.

“Immune Visibility Without Immune Efficacy” The IEM phenotype defies the conventional “hot vs. cold” classification (**Supplementary Table 1**). It generates what we define as “immune visibility without immune efficacy.” Operationally, this means the tumor appears immunologically “hot” based on standard clinical biomarkers (high PD-L1 Combined Positive Score [CPS] and dense immune infiltration - visibility), yet lacks the functionally competent, proliferating TCF1+ T-cell subpopulations and effective HLA-mediated antigen presentation required for cytotoxicity (lack of efficacy).

Falsifiable Predictions The utility of the IEM framework lies in its testable biological and therapeutic predictions:

Prediction 1: In prospective cohorts, we predict that a prespecified IEM-high profile will be associated with a lower objective response rate to PD-1 monotherapy than an IEM-low profile, after adjustment for PD-L1 status, disease burden, ART status, HIV viral suppression, and current and nadir CD4 counts (Evidence Tier: E4, hypothesis).
Prediction 2: We predict that longitudinal improvement in cervicovaginal community structure and epithelial barrier markers will correlate with reduced myeloid-inflammatory signaling and a lower terminal-to-progenitor exhausted T-cell ratio. Whether microbiome-directed intervention can causally produce these changes or improve checkpoint-blockade sensitivity requires prospective interventional testing (Evidence Tier: E4, hypothesis).

To clarify the structural mapping across these complex conceptual layers, we have integrated the baseline axes, HIV-induced perturbations, resulting IEM phenotype, and their clinical translation into a unified framework (**Table 5**).

**Table 5:** Integrative master mapping of the IEM framework in HIV-associated cervical cancer.

Baseline TIME architecture (section 4)	HIV-associated perturbation domain (section 5)	IEM phenotype component	Candidate multidimensional biomarkers (Section 7)	Prioritized therapeutic hypothesis (Section 6)
Axis 1: Effector & Antigen-Presenting Network	Domain 1 & 2: Compounded T-cell Exhaustion & Antigen-Presentation Impairment	“E” (Exhausted)	High TOX+/TCF1- T-cell ratio; HLA class I LOH; reduced cDC1 density.	Hypothesis 1: Combinatorial ICB (e.g., anti-PD-1 + anti-CTLA-4) or ICI + therapeutic vaccination.
Axis 2 & 4: Tolerogenic Niche & Mucosal Barrier	Domain 3 & 4: Myeloid-Regulatory Skewing & Persistent Mucosal Dysbiosis	“M” (Mucosally disrupted)	CST IV dominance (16S rRNA); elevated sCD14; high Treg/CD8+ ratio.	Hypothesis 2: Microenvironmental priming via defined live biotherapeutics or myeloid targeted therapies.
Axis 3: Co-inhibitory Network	Domain 5: Chronic Bystander Inflammation	“I” (Inflamed)	Elevated NLR, SII, LMR; High PD-L1 expression lacking functional T-cell infiltration.	Baseline ART optimization; continuous HIV-RNA monitoring; rigorous OI screening.

This integrative table clarifies the logical mapping between the baseline tumor immune microenvironment (TIME) axes, HIV-induced perturbations, the resulting IEM phenotype, and their translation into candidate biomarkers and mechanism-based testable hypotheses, reducing conceptual overlap across the manuscript.

ART, antiretroviral therapy; cDC1, conventional type 1 dendritic cell; CD8, cluster of differentiation 8; CST, community state type; CTLA-4, cytotoxic T-lymphocyte-associated protein 4; HLA, human leukocyte antigen; HIV, human immunodeficiency virus; ICB, immune checkpoint blockade; ICI, immune checkpoint inhibitor; IEM, inflamed, exhausted, and mucosally disrupted; LOH, loss of heterozygosity; LMR, lymphocyte-to-monocyte ratio; NLR, neutrophil-to-lymphocyte ratio; OI, opportunistic infection; PD-1, programmed cell death protein 1; PD-L1, programmed cell death ligand 1; RNA, ribonucleic acid; rRNA, ribosomal ribonucleic acid; sCD14, soluble CD14; SII, systemic immune-inflammation index; TCF1, T-cell factor 1; TIME, tumor immune microenvironment; TOX, thymocyte selection-associated high-mobility group box; Treg, regulatory T cell.

## 7 Therapeutic implications: prioritizing mechanism-based hypotheses for the IEM ecosystem

Translating the biology of the IEM phenotype into actionable clinical strategy requires a shift from uniform PD-1 blockade toward mechanism-stratified approaches (93). Specifically, the IEM phenotype establishes a profoundly immunosuppressive “cancer-immune set point” (94) that cannot be reversed by targeting a single axis. Given the limited direct evidence for novel immunotherapeutics in WLWH with cervical cancer, providing a broad catalogue of theoretical interventions risks overstating their current translational readiness. Instead, based on the proposed IEM framework, we prioritize two mechanism-based, experimentally testable therapeutic hypotheses, complemented by essential HIV-specific safety considerations.

### 7.1 Hypothesis 1: bypassing terminal exhaustion through combinatorial checkpoint blockade (targeting the “E” and “A” domains)

If the exhausted CD8(+) T-cell pool in HIV-associated tumors is heavily skewed toward a terminally exhausted (TOX-high/TCF1-low) state (Domain 1), single-agent PD-1 blockade is hypothesized to be insufficient.

Testable Hypothesis: Combinatorial regimens that target parallel inhibitory pathways (e.g., PD-1 plus CTLA-4 or TIGIT blockade) or combine immune checkpoint inhibitors (ICIs) with therapeutic HPV vaccines (7) (to overcome antigen-presentation failure and restore the critical CD4+ T-cell help essential for robust CD8+ T-cell cytotoxicity (95)) will yield superior objective response rates in IEM-high tumors compared to PD-1 monotherapy (93, 96).

### 7.2 Hypothesis 2: restoring mucosal-myeloid homeostasis to unlock ICI efficacy (targeting the “M” and “I” domains)

The active suppressive scaffold of the IEM Phenotype is sustained by mucosal barrier disruption (Domain 4) and subsequent myeloid-regulatory skewing (Domain 3).

Testable Hypothesis: Interventions aiming to restore cervicovaginal ecology (e.g., via defined live biotherapeutics, conceptually mirroring how microbiota interventions have overcome ICI resistance in melanoma (97, 98)), or utilize targeted anti-inflammatory agents (e.g., IL-6 blockade, which has been shown to decouple immune toxicity from anti-tumor efficacy (99)) prior to or alongside ICI initiation will attenuate maladaptive bystander inflammation (Domain 5). We hypothesize that this “microenvironmental priming” will reprogram the TIME from an IEM-high to an IEM-low state, thereby restoring sensitivity to standard ICI therapy.

Crucial Caveat: To our knowledge, no prospective clinical evidence currently demonstrates that microbiome-directed interventions improve antitumor efficacy, checkpoint-inhibitor response, or clinical outcomes in HIV-associated cervical cancer. This strategy should therefore be regarded as exploratory and hypothesis-generating.

### 7.3 HIV-specific safety, ART heterogeneity, and implementation barriers

Any immunotherapeutic strategy in WLWH must rigorously appraise clinical feasibility, prioritizing HIV-specific safety dynamics and the realities of high-burden settings (14, 15).

IRIS and HIV Reservoir Dynamics: ICI therapy presents uncommon but clinically relevant theoretical or reported risks, including Immune Reconstitution Inflammatory Syndrome (IRIS) or the unmasking of subclinical opportunistic infections. This necessitates baseline OI screening, CD4-guided prophylaxis, and infectious-disease consultation. Furthermore, while ICIs may enhance HIV-specific T-cell activity or induce transient HIV transcription/latency reversal, their clinical impact on reservoir size remains unproven. Trials should incorporate longitudinal monitoring of HIV RNA and CD4+ counts, and may include cell-associated HIV DNA/RNA as an exploratory endpoint, without implying that ICIs will eradicate the HIV reservoir.

ART Heterogeneity and Drug-Drug Interactions (DDIs): ART regimens are not biologically uniform. For instance, integrase strand transfer inhibitors (INSTIs, e.g., dolutegravir) may drive metabolic and inflammatory profiles differing markedly from those of non-nucleoside reverse transcriptase inhibitors (NNRTIs) or protease inhibitors (PIs) (37, 62). Regarding DDIs, direct pharmacokinetic interactions between ART and monoclonal-antibody ICIs are expected to be limited because these antibodies are cleared predominantly through proteolytic catabolism. However, clinically relevant interactions may arise with concomitant chemotherapy, antibody–drug conjugates, small-molecule agents, corticosteroids, or anti-infective therapies (e.g., CYP3A4-mediated interactions involving ritonavir/cobicistat, efavirenz, or rifampicin) (100).

Implementation Barriers in LMICs: Translating the IEM framework into clinical practice in low- and middle-income countries (LMICs)—where the HIV-cervical cancer co-burden is concentrated—faces substantial structural barriers (13, 46). The requirement for repeated cervical biopsies to validate dynamic spatial and single-cell biomarker panels presents severe logistical, financial, and acceptability challenges. Consequently, advanced multidimensional biomarker panels may currently lack cost-effectiveness and feasibility in these settings. This underscores the urgent need to validate minimally invasive, blood-based exploratory early-response biomarker endpoints, such as HPV-ctDNA kinetics, tailored specifically for resource-constrained environments (101, 102).

## 8 Biomarker landscapes: candidate multidimensional coordinates for the IEM ecosystem

A central challenge in developing biomarker-informed immunotherapy for WLWH with cervical cancer is that the performance and biological interpretation of conventional biomarkers have not been adequately evaluated in this population. Biomarkers used or investigated in cervical cancer and other solid tumors—including PD-L1 Combined Positive Score (CPS), less commonly Tumor Proportion Score, and tumor mutational burden—were largely validated in cohorts with limited representation of WLWH. Chronic HIV-associated immune activation, heterogeneous immune reconstitution during ART, persistent interferon signalling, and mucosal inflammation could influence their interpretation, although the direction and clinical magnitude of these effects remain uncertain (19, 96).

PD-L1 expression may reflect adaptive immune resistance associated with tumor-reactive T-cell activity, but it may also be induced by bystander interferon exposure, myeloid activation, infection, or tissue injury. These limitations are not unique to HIV, although their relative contribution may differ in some WLWH. PD-L1 should therefore not be interpreted as a standalone measure of productive antitumor immunity. This does not negate its indication-specific clinical use, and validated PD-L1 testing should continue where required. Similarly, TMB does not directly quantify HPV-derived antigenicity, antigen-presentation competence, or antigen-specific T-cell function, and its predictive performance in WLWH with cervical cancer remains undefined.

The proposed IEM framework supports investigation of multidimensional and longitudinal biomarker combinations rather than reliance on a single static threshold. Candidate biomarkers should be classified according to their intended context of use: prognostic, treatment-predictive, pharmacodynamic, response-monitoring, safety-related, or mechanistic. These functions should not be assumed to be interchangeable. Candidate biomarkers aligned with the five biological domains are summarized in **Table 6**.

**Table 6:** Operationalization of the proposed five-domain IEM framework.

Proposed domain	Core alteration	Candidate biomarkers	Potential translational relevance	Evidence status
Compounded T-cell exhaustion	Persistent HPV antigen exposure superimposed on HIV-associated T-cell dysfunction	PD-1, TIM-3, LAG-3, TIGIT, TOX, TCF1; HPV-specific T-cell responses	Characterization of dysfunctional T-cell states and potential resistance to checkpoint blockade	Supported by HIV and cervical cancer studies; direct evidence in HIV-associated cervical tumors remains limited
Antigen-presentation impairment	Defective dendritic-cell function and reduced tumor-antigen processing or presentation	cDC1 density; HLA-I, β2M, TAP1/2; BATF3 and cross-presentation signatures	Identification of tumors that may require restoration of immune priming or antigen presentation	Predominantly supported by indirect or extrapolated evidence
Myeloid-regulatory skewing	Enrichment of suppressive macrophage, MDSC, and Treg programs	CD163, ARG1, IDO1, FOXP3, CD39/CD73; kynurenine-to-tryptophan ratio	Evaluation of myeloid- or regulatory-cell-mediated immune suppression	Biologically plausible, but HIV-specific tumor validation is needed
Mucosal disruption and dysbiosis	Lactobacillus depletion, epithelial barrier injury, and altered local immune signaling	Cervicovaginal community state type; barrier markers; local cytokine and metabolite profiles	Assessment of host–microbiome interactions and mucosal inflammatory states	Supported by mucosal and microbiome studies; tumor-specific causality is unresolved
Chronic bystander inflammation	Persistent interferon and inflammatory signaling without effective tumor-cell killing	IFN-response signatures; IL-6, CXCL9/10/11, CRP, sCD14, and LBP	Distinguishing productive antitumor inflammation from nonspecific immune activation	Strong HIV-related evidence, but limited validation in HIV-associated cervical cancer

The IEM framework and listed biomarkers are proposed for research validation and should not be interpreted as an established molecular classification or clinical decision tool.

ARG1, arginase 1; ART, antiretroviral therapy; BATF3, basic leucine zipper ATF-like transcription factor 3; β2M, beta-2-microglobulin; cDC1, conventional type 1 dendritic cell; CRP, C-reactive protein; CXCL, C-X-C motif chemokine ligand; FOXP3, forkhead box P3; HLA-I, human leukocyte antigen class I; HPV, human papillomavirus; IDO1, indoleamine 2, 3-dioxygenase 1; IEM, inflamed, exhausted, and mucosally disrupted; IFN, interferon; IL-6, interleukin 6; LAG-3, lymphocyte-activation gene 3; LBP, lipopolysaccharide-binding protein; MDSC, myeloid-derived suppressor cell; PD-1, programmed cell death protein 1; sCD14, soluble CD14; TAP1/2, transporter associated with antigen processing 1 and 2; TCF1, T-cell factor 1; TIGIT, T-cell immunoreceptor with immunoglobulin and ITIM domains; TIM-3, T-cell immunoglobulin and mucin-domain containing 3; TOX, thymocyte selection-associated high-mobility group box; Treg, regulatory T cell.

### 8.1 Domain-aligned biomarker layers

Systemic and local inflammation. Candidate measures include NLR, SII, and LMR (90). Chronic lymphopenia and myeloid inflammation may be associated with higher NLR and SII and lower LMR. However, these indices are nonspecific and can be influenced by active infection, corticosteroids, growth-factor support, bone-marrow suppression, tumor burden, and recent anticancer treatment. We hypothesize that elevated baseline or persistently elevated on-treatment inflammatory indices may be associated with suppressive intratumoral immune states. Whether such measures are prognostic or specifically predictive of reduced ICI benefit requires treatment-by-biomarker interaction analyses. Initial analyses should retain these variables as continuous measures, with candidate thresholds requiring external validation.

T-cell state and exhaustion architecture. Candidate measures include co-expression patterns of PD-1, TIM-3, TIGIT, and LAG-3 on CD8^+^ TILs; TOX as a marker associated with exhaustion-lineage programming; and the abundance and spatial distribution of TCF1-positive progenitor-like and more differentiated TCF1-low/negative CD8^+^ T-cell states (92, 93, 103). Inhibitory-receptor expression should be interpreted together with transcriptional state, proliferation, effector competence, clonotype, and spatial localization. TCR repertoire measures—including diversity, clonality, tumor–blood overlap, and treatment-emergent clonal expansion—may provide complementary information, but no uniform direction of association with response should be assumed (104). TCR sequencing alone does not establish HPV or tumor specificity.

Antigen-presentation integrity. Candidate measures include HLA class I loss of heterozygosity inferred from validated tumor–normal sequencing pipelines, tumor-cell β2-microglobulin expression and spatial heterogeneity, expression of antigen-processing genes, and intratumoral cDC1 abundance identified using markers such as CLEC9A and XCR1 (105). The hypothesis that cDC1 abundance is reduced in cervical tumors from WLWH requires direct validation in appropriately matched cohorts.

Myeloid and regulatory-cell composition. Candidate measures include the FOXP3^+^/CD8^+^ ratio, spatially resolved macrophage states, peripheral MDSC frequency, and selected circulating metabolic markers (106). Macrophages should be analyzed as heterogeneous functional states rather than through a binary M1:M2 classification. The kynurenine-to-tryptophan ratio should be interpreted as a nonspecific measure of systemic tryptophan catabolism rather than direct tumor IDO1 activity. MDSC, ARG1, and adenosine-pathway measurements require standardized sample processing and should not be assumed to reflect intratumoral activity.

Mucosal ecology and barrier-associated measures. Candidate biomarkers include cervicovaginal community composition assessed by standardized 16S rRNA sequencing or metagenomics, the relative abundance of Lactobacillus-dominant and anaerobe-enriched communities, local inflammatory mediators, and direct or indirect barrier-associated measures (35, 39). Systemic sCD14 and I-FABP are nonspecific markers associated with microbial-product exposure or intestinal epithelial injury and do not localize barrier dysfunction to the cervicovaginal tract. Composite microbiome–inflammation scores may be developed as exploratory research biomarkers, but no threshold has been validated for treatment selection or microbiome-directed intervention in cervical cancer.

### 8.2 Dynamic biomarkers: liquid biopsy and HPV-ctDNA

Because cervical tumors may be spatially heterogeneous and repeated tissue sampling can be impractical, liquid biopsy provides a complementary longitudinal approach. Circulating tumor HPV DNA, measured within plasma cell-free DNA, is a promising response-monitoring biomarker in HPV-driven cancers (101, 102). Decline or clearance at prespecified early on-treatment time points may correlate with tumor response, but HPV-ctDNA has not been validated as a surrogate endpoint for survival or as a basis for treatment modification in WLWH with cervical cancer.

Assays should distinguish tumor-derived HPV DNA from background anogenital HPV detection, particularly in patients with persistent or multiple HPV infections. HPV-ctDNA should be interpreted primarily as a tumor-burden or response marker rather than a direct measure of immune-mechanism reversal. Tumor-informed nonviral ctDNA dynamics may provide an orthogonal measure, provided that germline variants and clonal hematopoiesis are appropriately excluded.

Additional longitudinal measures include circulating immune-cell phenotyping and plasma cytokines such as IL-6, IL-8, sCD163, and CXCL10. These measures are nonspecific and should be interpreted in relation to HIV RNA, intercurrent infection, corticosteroid or growth-factor exposure, and anticancer-treatment timing. Liquid biopsy is minimally invasive and suitable for HIV-inclusive trials, but its scalability in LMIC settings will depend on assay cost, sample stabilization, laboratory capacity, and external quality assurance.

### 8.3 Spatial and integrative biomarkers

Spatial and single-cell technologies may improve mechanistic resolution of the proposed IEM framework (107). Multiplex immunofluorescence and imaging mass cytometry can characterize the spatial relationships among tumor cells, T-cell states, myeloid populations, and HPV-associated tumor epithelium. Spatial transcriptomic approaches may map exhaustion, interferon, antigen-presentation, and stromal programs within their tissue context (108). Paired single-cell RNA and TCR sequencing can identify clonal T-cell states and treatment-associated expansion, although antigen specificity requires complementary functional or peptide–HLA-based validation (109).

Tissue site, prior treatment, fixation, tumor cellularity, necrosis, and batch effects should be standardized or recorded because they may materially influence spatial readouts. In the longer term, integrative models combining clinical variables, inflammatory indices, ctDNA kinetics, microbiome measures, and spatial features could be developed as quantitative research classifiers (110). Clinical implementation would require analytical validation, independent external validation, calibration, assessment of transportability across geographically and ancestrally diverse populations, and prospective demonstration of incremental clinical utility.

## 9 A two-dimensional research stratification framework for future clinical trials

The historical exclusion of WLWH from immunotherapy trials necessitates the development of inclusive, biologically informed trial designs. However, systemic HIV control and the local tumor immune phenotype are related but non-equivalent dimensions. For example, a patient may achieve systemic virological suppression and CD4+ T-cell reconstitution under ART, yet persistently harbor a smoldering, exhausted (IEM-high) cervical TIME.

To transition the IEM framework into a concrete trial-design tool, we propose shifting from unidimensional CD4-based stratification to a two-dimensional (2D) research stratification matrix (**Table 7**). This matrix cross-tabulates systemic HIV/immune status with the local tumor IEM phenotype, aiming to objectively inform the following eight core domains of prospective clinical trial design:

**Table 7:** Proposed two-dimensional (2D) research stratification matrix for HIV-associated cervical cancer trials.

Local tumor TIME \ systemic HIV & immune status	Systemic HIV & immune status: controlled (HIV RNA suppressed, CD4 >200 cells/μL, no active OIs)	Systemic HIV & immune status: unstable/immunocompromised (HIV RNA detectable, or CD4 <200 cells/μL, or recent OIs)
Local Tumor TIME: IEM-Low(Classical Hot or Cold, conventional T-cell subsets, CST I/II/III)	Quadrant I (Standard Risk/Standard Biology):Hypothesized to behave similarly to HIV-negative cohorts. Potential candidates for standard-of-care ICI regimens.	Quadrant II (High Risk/Standard Biology):Toxicity and infection risk elevated. Requires strict ART optimization and intensive safety monitoring during ICI therapy.
Local Tumor TIME: IEM-High(Severe chronic inflammation, terminal TOX+ exhaustion, CST IV dysbiosis)	Quadrant III (Standard Risk/Altered Biology):Systemically stable but locally resistant. Prime candidates for trials investigating mechanism-matched combinatorial therapies (e.g., dual-ICB, mucosal modulation).	Quadrant IV (High Risk/Altered Biology):Highest complexity. Exploratory cohorts only. Focus on safety, ART optimization, and reversal of baseline myeloid/mucosal inflammation prior to aggressive ICB.

This 2D matrix conceptually separates systemic virological control from the local tumor immune phenotype, providing a structured framework for defining eligibility, stratification, and treatment allocation in future prospective trials.

2D, two-dimensional; ART, antiretroviral therapy; CST, community state type; HIV, human immunodeficiency virus; ICB, immune checkpoint blockade; ICI, immune checkpoint inhibitor; IEM, inflamed, exhausted, and mucosally disrupted; OIs, opportunistic infections; RNA, ribonucleic acid; TIME, tumor immune microenvironment; TOX, thymocyte selection-associated high-mobility group box.

1. Eligibility Criteria: Standard criteria typically mandate strict CD4+ thresholds (e.g., >200 cells/μL) and undetectable HIV RNA. In an IEM-informed framework, eligibility could be broadened. Patients with stable but lower CD4+ counts could be eligible for inclusion in dedicated exploratory cohorts, provided active opportunistic infections (OIs) are strictly excluded, as their local tumor immunity may still retain targetable subsets.
2. HIV-Related Stratification Factors: Rather than randomizing patients globally, block randomization should be applied using the Two-Dimensional (2D) Research Stratification Grid. This ensures that treatment arms are balanced by Systemic HIV Status (Controlled vs. Incompletely controlled) and Tumor IEM Status (High vs. Low). Systemic Immune Status must be defined using explicit, though flexible, numerical thresholds (e.g., >500, 200–500, and <200 cells/μL). However, these cross-sectional values must be interpreted strictly with the caveat of the patient’s historical CD4+ nadir and the temporal trajectory of immune reconstitution, rather than as rigid standalone parameters. Current CD4+ count, ART duration, and these dynamic immunological metrics must be fully integrated as essential sub-strata.
3. Treatment Allocation: The 2D framework provides a mechanistic rationale for differential investigation. Importantly, IEM status should initially be used as a prespecified stratification or enrichment variable within randomized hypothesis-testing trials, rather than as a validated basis for routine treatment assignment.
4. Prospective Biomarker-Defined Cohorts: Trials should include exploratory biomarker-defined parallel cohorts based on the specific dominance of IEM features for prospective validation. Optimally, this should include an HIV-negative comparator cohort to rigorously establish the specificity of the HIV-associated TIME perturbations.
5. Sampling Schedules: Because the IEM phenotype emphasizes the dynamic interplay between HIV, HPV, and the TIME, robust biological sampling is required. Protocols should mandate baseline tumor biopsies alongside serial blood (e.g., HPV-ctDNA, HIV RNA, CD4+) and cervicovaginal sampling. However, acknowledging the severe logistical and financial barriers of repeated tissue biopsies in low- and middle-income countries (LMICs), on-treatment or progression biopsies should be incorporated as optional or where clinically feasible.
6. Primary and Secondary Endpoints: While standard endpoints (ORR and PFS) remain primary, IEM-informed trials should incorporate biologically specific endpoints. The kinetic clearance of HPV-ctDNA should be evaluated as an exploratory early-response biomarker endpoint for immune efficacy, alongside quantitative shifts in cervicovaginal dysbiosis scores serving as an exploratory translational endpoint.
7. Safety Monitoring: Safety protocols in WLWH must uniquely differentiate between Immune-Related Adverse Events (irAEs) and uncommon but clinically relevant theoretical risks, such as HIV Immune Reconstitution Inflammatory Syndrome (IRIS). Intensive baseline screening and longitudinal monitoring for OIs, HBV/HCV status, CD4+ count, and HIV RNA are mandatory, coupled with appropriate, CD4-guided OI prophylaxis.
8. Analysis of Treatment Interactions with ART: ART optimization is the foundational pharmacological platform. Trial analyses must prospectively capture interactions based on specific ART classes (e.g., INSTIs, NNRTIs, boosted PIs) rather than treating ART as a uniform variable. Statistical models must account for concomitant pharmacologic boosters or anti-infectives (e.g., ritonavir/cobicistat, rifampicin) to isolate the true efficacy of the oncological intervention from ART-associated immune recovery or pharmacokinetic alterations.

## 10 Overcoming translational chasms: next-generation preclinical models

Despite emerging evidence supporting the feasibility of immunotherapy in selected people living with HIV, mechanistic data specific to HIV-associated cervical cancer remain limited. Model development is complicated by HIV and HPV species tropism, the prolonged natural history of HPV-driven carcinogenesis, HLA-restricted immunity, ART exposure, and the difficulty of reproducing the human cervicovaginal microenvironment and microbiota (111). Conventional murine models capture selected aspects of HPV oncogenesis or antitumor immunity but do not reproduce the full host-mediated interaction among HIV, HPV-associated cancer, systemic immunity, and mucosal ecology.

Humanized mouse models, including BLT and selected myeloid-supportive platforms, may reproduce specific features of HIV infection, immune perturbation, ART exposure, and treatment pharmacodynamics (110). However, incomplete immune reconstitution, HLA mismatch, graft-versus-host disease, nonphysiological lymphoid architecture, limited microbiome fidelity, and species-specific pharmacology constrain their interpretation. Model selection should therefore be driven by the biological question rather than by an assumption that any single platform recapitulates the IEM phenotype.

Complementary ex vivo systems should include patient-derived organoids, short-term tumor explants, and multicellular co-cultures incorporating autologous immune and stromal components where feasible (112). Controlled HIV-related perturbations may be introduced to investigate defined mechanisms, but simple co-culture with HIV-infected allogeneic PBMCs may generate nonphysiological effects and requires stringent biosafety procedures. Microphysiological cervicovaginal systems may enable investigation of selected epithelial–immune–microbial interactions, particularly when they incorporate relevant epithelial architecture, stromal and immune cells, hormonal conditions, oxygen gradients, and defined microbial communities (113). These platforms nevertheless remain reductionist and cannot yet reproduce the complete mucosal ecology of WLWH.

We therefore propose a question-driven and iterative model portfolio rather than a fixed linear pipeline: organoids and tumor explants for candidate screening and tissue pharmacodynamics; microphysiological systems for barrier and microbial cross-talk; and humanized *in vivo* models for systemic pharmacology, mechanistic evaluation, and preliminary safety signals. Findings should be iteratively compared with clinical samples and observational data before translation into carefully staged early-phase or investigator-initiated studies. No preclinical platform should be considered confirmatory of clinical efficacy.

## 11 Limitations and unanswered questions

While the proposed IEM framework provides a structured approach to understanding the HIV-associated cervical TIME, it is essential to clearly delineate its current limitations and the critical questions that remain unanswered:

Lack of Direct Mechanistic Validation: The most significant limitation of the IEM framework is its reliance on extrapolated evidence. Currently, several proposed mechanisms—particularly the cross-compartmental signaling between HIV-infected stroma and HPV-driven epithelium—are derived from *in vitro* studies, preclinical models, or inferred from parallel fields (e.g., HIV-negative cervical cancer and non-tumor HIV immunology). Direct, spatially resolved validation within intact human HIV-associated cervical tumors remains sparse.

Specificity and the “Continuum” Concept: It remains an unanswered question whether the IEM phenotype is exclusively enriched among WLWH. Rather than a binary HIV-specific state, IEM may represent a biological continuum that overlaps with cervical cancer in HIV-negative women experiencing severe immune suppression from other etiologies (e.g., chronic systemic inflammation, malnutrition, or advanced age). Future comparative cohorts must determine the true specificity of this phenotype.

Predictive Limitations: At its current stage of development, the IEM framework is a hypothesis-generating tool for biological stratification; it cannot yet reliably predict individual clinical responses or toxicities. It remains unknown precisely which quantitative threshold of exhaustion (e.g., specific TOX+/TCF1- ratios) or which degree of mucosal dysbiosis definitively precludes ICI benefit.

Cost-effectiveness and Feasibility in LMICs: The high-resolution validation of the IEM phenotype relies heavily on advanced single-cell sequencing, spatial transcriptomics, and multidimensional biomarker panels. These technologies are currently neither cost-effective nor logistically feasible for routine implementation in low- and middle-income countries (LMICs), where the disease burden is overwhelmingly concentrated. Bridging the gap between high-dimensional discovery and accessible, cost-effective clinical assays (e.g., targeted blood-based surrogate markers) remains a monumental challenge.

Ultimately, the IEM framework highlights our conceptual blind spots and sets a roadmap for targeted investigation, but it requires rigorous prospective validation before it can definitively guide clinical decision-making.

## 12 Conclusion and future perspectives

As immuno-oncology rapidly expands, its transformative benefits must be rigorously evaluated in populations historically underrepresented in therapeutic development. WLWH with cervical cancer should no longer be viewed simply as a high-risk demographic to be excluded from immunotherapy trials due to perceived vulnerabilities. They constitute a clinically and immunologically heterogeneous population requiring inclusive, prospectively designed studies and context-sensitive trial frameworks.

The proposed IEM framework seeks to provide a shared language for integrating HIV medicine and immuno-oncology. By acknowledging that HIV-associated cervical tumors may occupy a chronically inflamed yet functionally constrained immune state, this framework underscores the inadequacy of simple “hot-versus-cold” tumor classifications and unidimensional CD4+ thresholds.

Moving forward, the priority must shift from retrospective extrapolation to prospective evidence generation. Future WLWH-enriched cohorts must incorporate standardized longitudinal HIV and immune monitoring, paired tissue and liquid biopsy sampling, and guideline-concordant ART management directly into oncology protocols. Crucially, these research programs must be equitably co-designed and co-led by investigators and communities in regions bearing the highest HIV–cervical cancer co-burden, such as sub-Saharan Africa.

The central question for the next decade is no longer simply whether WLWH can safely receive immunotherapy, but how benefit, risk, and optimal combinatorial strategies vary across the spectrum of HIV-related immune states—and how that knowledge can be translated rigorously and equitably to the patients who need it most.

## Glossary

APC: antigen-presenting cell
ARG1: arginase 1
ART: antiretroviral therapy
β2M: beta-2-microglobulin
CC: cervical cancer
cDC1: conventional type 1 dendritic cell
CI: confidence interval
CIN: cervical intraepithelial neoplasia
CPS: combined positive score
CRT: chemoradiotherapy
CST: community state type
CTLA-4: cytotoxic T-lymphocyte-associated protein 4
ctDNA: circulating tumor DNA
CTL: cytotoxic T lymphocyte
CXCL: C-X-C motif chemokine ligand
CYP: cytochrome P450
DDI: drug-drug interaction
DNMT: DNA methyltransferase
EV: extracellular vesicle
FIGO: International Federation of Gynecology and Obstetrics
Flt3L: FMS-like tyrosine kinase 3 ligand
HDAC: histone deacetylase
HIV: human immunodeficiency virus
HLA: human leukocyte antigen
HLA-LOH: human leukocyte antigen loss-of-heterozygosity
HPV: human papillomavirus
hrHPV: high-risk human papillomavirus
HR: hazard ratio
HSIL: high-grade squamous intraepithelial lesion
ICB: immune checkpoint blockade
ICI: immune checkpoint inhibitor
IDO1: indoleamine 2, 3-dioxygenase 1
IEM: Inflamed, Exhausted, and mucosally disrupted
I-FABP: intestinal fatty acid-binding protein
IFN-γ: interferon gamma
IHC: immunohistochemistry
IIT: investigator-initiated trial
IL: interleukin
INSTI: integrase strand transfer inhibitor
irAE: immune-related adverse event
IRIS: immune reconstitution inflammatory syndrome
ITT: intention-to-treat
JAK: Janus kinase
LAG-3: lymphocyte-activation gene 3
LBP: lipopolysaccharide-binding protein
LCR: long control region
LMIC: low- and middle-income countries
LMR: lymphocyte-to-monocyte ratio
LPS: lipopolysaccharide
MDSC: myeloid-derived suppressor cell
MTB: multidisciplinary tumor board
NGS: next-generation sequencing
NK: natural killer
NLR: neutrophil-to-lymphocyte ratio
ORR: objective response rate
OI: opportunistic infection
OS: overall survival
PBMC: peripheral blood mononuclear cell
PD-1: programmed cell death protein 1
PD-L1: programmed death-ligand 1
PDO: patient-derived organoid
PFS: progression-free survival
PLWH: people living with HIV
sCD14: soluble CD14
SII: systemic immune-inflammation index
STING: stimulator of interferon genes
TAM: tumor-associated macrophage
TAP1: transporter associated with antigen processing 1
TCF1: T cell factor 1
TCR: T-cell receptor
TGF-β: transforming growth factor beta
TIGIT: T cell immunoreceptor with Ig and ITIM domains
TIL: tumor-infiltrating lymphocyte
TIM-3: T-cell immunoglobulin and mucin-domain containing-3
TIME: tumor immune microenvironment
TMB: tumor mutational burden
TOX: thymocyte selection-associated high mobility group box
TPS: tumor proportion score
Treg: regulatory T cell
TRM: tissue-resident memory T cell
VMT: vaginal microbiome transplantation
WHO: World Health Organization
WLWH: women living with HIV

## References

[1] Colombo N Dubot C Lorusso D Cibula D Mirza MR Ryu HS . Pembrolizumab for persistent, recurrent, or metastatic cervical cancer. N Engl J Med. (2021) 385:1856–67. doi: 10.1056/NEJMoa2112435 34534429

[2] Tewari KS Monk BJ Vergote I Miller A de Melo AC Kim HS . Survival with cemiplimab in recurrent cervical cancer. N Engl J Med. (2022) 386:544–55. doi: 10.1056/NEJMoa2112187 35139273

[3] Oaknin A Gladieff L Martínez-García J Villacampa G Takekuma M De Giorgi U . Atezolizumab plus bevacizumab and chemotherapy for metastatic, persistent, or recurrent cervical cancer (BEATcc): a randomised, open-label, phase 3 trial. Lancet. (2024) 403:31–43. doi: 10.1016/S0140-6736(23)02405-4 38048793

[4] Lorusso D Xiang Y Hasegawa K Scambia G Leiva M Ramos-Elias P . Pembrolizumab or placebo with chemoradiotherapy followed by pembrolizumab or placebo for newly diagnosed, high-risk, locally advanced cervical cancer (ENGOT-cx11/GOG-3047/KEYNOTE-A18): a randomised, double-blind, phase 3 clinical trial. Lancet. (2024) 403:1341–50. doi: 10.1016/S0140-6736(24)00317-9 38521086

[5] Zur Hausen H . Papillomaviruses and cancer: from basic studies to clinical application. Nat Rev Cancer. (2002) 2:342–50. doi: 10.1038/nrc798 12044010

[6] Khan I Harshithkumar R More A Mukherjee A . Human papilloma virus: an unraveled enigma of universal burden of Malignancies. Pathogens. (2023) 12:564. doi: 10.3390/pathogens12040564 37111450 PMC10146077

[7] Hancock G Hellner K Dorrell L . Therapeutic HPV vaccines. Best Pract Res Clin Obstet Gynaecol. (2018) 47:59–72. doi: 10.1016/j.bpobgyn.2017.09.008 29108943

[8] Dey T Agrawal S . Immunotherapy in cervical cancer: an innovative approach for better treatment outcomes. Explor Target Antitumor Ther. (2025) 6:1002296. doi: 10.37349/etat.2025.1002296 40061136 PMC11886377

[9] Bray F Laversanne M Sung H Ferlay J Siegel RL Soerjomataram I . Global cancer statistics 2022: GLOBOCAN estimates of incidence and mortality worldwide for 36 cancers in 185 countries. CA Cancer J Clin. (2024) 74:229–63. doi: 10.3322/caac.21834 38572751

[10] Stelzle D Tanaka LF Lee KK Ibrahim Khalil A Baussano I Shah ASV . Estimates of the global burden of cervical cancer associated with HIV. Lancet Glob Health. (2021) 9:e161–9. doi: 10.1016/S2214-109X(20)30459-9 33212031 PMC7815633

[11] Denslow SA Rositch AF Firnhaber C Ting J Smith JS . Incidence and progression of cervical lesions in women with HIV: a systematic global review. Int J STD AIDS. (2014) 25:163–77. doi: 10.1177/0956462413491735 24216030 PMC5524184

[12] Swinkels HM Nguyen AD Samandari T Gulick PG . HIV and AIDS. In: Statpearls. StatPearls Publishing, Treasure Island (FL (2026). 30521281

[13] Klutse J Tay YA Attoh DA Gyekye EF Bortey CB Buatsi ED . Cervical cancer in women with HIV: a call to action for equitable prevention in low- and middle-income countries. BioMed Res Int. (2025) 2025:1711050. doi: 10.1155/bmri/1711050 41367932 PMC12683070

[14] Persad GC Little RF Grady C . Including persons with HIV infection in cancer clinical trials. J Clin Oncol. (2008) 26:1027–32. doi: 10.1200/JCO.2007.14.5532 18309938

[15] Uldrick TS Ison G Rudek MA Noy A Schwartz K Bruinooge S . Modernizing clinical trial eligibility criteria: recommendations of the American Society of Clinical Oncology-Friends of Cancer Research HIV Working Group. J Clin Oncol. (2017) 35:3774–80. doi: 10.1200/JCO.2017.73.7338 28968173 PMC5793223

[16] Konopnicki D Manigart Y Gilles C Barlow P de Marchin J Feoli F . Sustained viral suppression and higher CD4+ T-cell count reduces the risk of persistent cervical high-risk human papillomavirus infection in HIV-positive women. J Infect Dis. (2013) 207:1723–9. doi: 10.1093/infdis/jit090 23463709

[17] Kelly H Weiss HA Benavente Y de Sanjose S Mayaud P ART and HPV Review Group . Association of antiretroviral therapy with high-risk human papillomavirus, cervical intraepithelial neoplasia, and invasive cervical cancer in women living with HIV: a systematic review and meta-analysis. Lancet HIV. (2018) 5:e45–58. doi: 10.1016/S2352-3018(17)30149-2 29107561 PMC5757426

[18] Liu G Sharma M Tan N Barnabas RV . HIV-positive women have higher risk of human papilloma virus infection, precancerous lesions, and cervical cancer. AIDS. (2018) 32:795–808. doi: 10.1097/QAD.0000000000001765 29369827 PMC5854529

[19] Swase TD Fasogbon IV Eseoghene IJ Etukudo EM Mbina SA Joan C . The impact of HPV/HIV co-infection on immunosuppression, HPV genotype, and cervical cancer biomarkers. BMC Cancer. (2025) 25:202. doi: 10.1186/s12885-025-13516-2 39910495 PMC11796042

[20] Anahtar MN Byrne EH Doherty KE Bowman BA Yamamoto HS Soumillon M . Cervicovaginal bacteria are a major modulator of host inflammatory responses in the female genital tract. Immunity. (2015) 42:965–76. doi: 10.1016/j.immuni.2015.04.019 25992865 PMC4461369

[21] Klatt NR Cheu R Birse K Zevin AS Perner M NoëlRomas L . Vaginal bacteria modify HIV tenofovir microbicide efficacy in African women. Science. (2017) 356:938–45. doi: 10.1126/science.aai9383 28572388

[22] Pavone G Marino A Fisicaro V Motta L Spata A Martorana F . Entangled connections: HIV and HPV interplay in cervical cancer-a comprehensive review. Int J Mol Sci. (2024) 25:10358. doi: 10.3390/ijms251910358 39408687 PMC11477307

[23] Guo W Dai L Qiu L . T cell subsets in cervical cancer tumor microenvironment: advances and therapeutic opportunities. Front Immunol. (2025) 16:1612032. doi: 10.3389/fimmu.2025.1612032 40539072 PMC12176900

[24] Omar A Marques N Crawford N . Cancer and HIV: the molecular mechanisms of the deadly duo. Cancers (Basel). (2024) 16:546. doi: 10.3390/cancers16030546 38339297 PMC10854577

[25] Obanya DI Wootton LM Morgan EL . Advances in understanding the mechanisms of the human papillomavirus oncoproteins. Biochem Soc Trans. (2025) 53:565–77. doi: 10.1042/BST20253041 40380881 PMC12224896

[26] Karnik M Tulimilli SV Anantharaju PG Bettadapura ADS Natraj SM Mohideen HS . An overview of the mechanisms of HPV-induced cervical cancer: the role of kinase targets in pathogenesis and drug resistance. Cancers (Basel). (2026) 18:318. doi: 10.3390/cancers18020318 41595237 PMC12838760

[27] Li Y Deng J Liu Y Yu S . HPV infection and the immune microenvironment in cervical cancer. Front Immunol. (2025) 16:1645019. doi: 10.3389/fimmu.2025.1645019 40808954 PMC12343561

[28] Li T Zhang H Lian M He Q Lv M Zhai L . Global status and attributable risk factors of breast, cervical, ovarian, and uterine cancers from 1990 to 2021. J Hematol Oncol. (2025) 18:5. doi: 10.1186/s13045-025-01660-y 39794860 PMC11721161

[29] World Health Organization . Global Strategy to Accelerate the Elimination of Cervical Cancer as a Public Health Problem. Geneva: WHO (2020).

[30] McDonald AC Tergas AI Kuhn L Denny L Wright TC Jr. Distribution of human papillomavirus genotypes among HIV-positive and HIV-negative women in Cape Town, South Africa. Front Oncol. (2014) 4:48. doi: 10.3389/fonc.2014.00048 24672770 PMC3953716

[31] Taku O Mbulawa ZZA Phohlo K Garcia-Jardon M Businge CB Williamson AL . Distribution of human papillomavirus (HPV) genotypes in HIV-negative and HIV-positive women with cervical intraepithelial lesions in the Eastern Cape Province, South Africa. Viruses. (2021) 13:280. doi: 10.3390/v13020280 33670231 PMC7916956

[32] Wilkin TJ Chen H Cespedes MS LeonCruz JT Godfrey C Chiao EY . A randomized, placebo-controlled trial of the quadrivalent human papillomavirus vaccine in human immunodeficiency virus-infected adults aged 27 years or older: AIDS Clinical Trials Group Protocol A5298. Clin Infect Dis. (2018) 67:1339–46. doi: 10.1093/cid/ciy274 29659751 PMC6186857

[33] Grover S BvochoraNsingo M Yeager A Chiyapo S Bhatia R MacDuffie E . Impact of human immunodeficiency virus infection on survival and acute toxicities from chemoradiation therapy for cervical cancer patients in a limited-resource setting. Int J Radiat Oncol Biol Phys. (2018) 101:201–10. doi: 10.1016/j.ijrobp.2018.01.067 29619965 PMC5999036

[34] Ferreira MP Coghill AE Chaves CB Bergmann A Thuler LC Soares EA . Outcomes of cervical cancer among HIV-infected and HIV-uninfected women treated at the Brazilian National Institute of Cancer. AIDS. (2017) 31:523–31. doi: 10.1097/QAD.0000000000001367 28060014 PMC5263104

[35] Brenchley JM Douek DC . Microbial translocation across the GI tract. Annu Rev Immunol. (2012) 30:149–73. doi: 10.1146/annurev-immunol-020711-075001 22224779 PMC3513328

[36] Mehandru S Poles MA TennerRacz K Horowitz A Hurley A Hogan C . Primary HIV-1 infection is associated with preferential depletion of CD4+ T lymphocytes from effector sites in the gastrointestinal tract. J Exp Med. (2004) 200:761–70. doi: 10.1084/jem.20041196 15365095 PMC2211967

[37] Deeks SG Overbaugh J Phillips A Buchbinder S . HIV infection. Nat Rev Dis Primers. (2015) 1:15035. doi: 10.1038/nrdp.2015.35 27188527

[38] Loharamtaweethong K Vinyuvat S Thammasiri J Chitpakdee S Supakatitham C Puripat N . Impact of antiretroviral drugs on PD-L1 expression and copy number gains with clinical outcomes in HIV-positive and -negative locally advanced cervical cancers. Oncol Lett. (2019) 18:5747–58. doi: 10.3892/ol.2019.10963 31788048 PMC6868352

[39] Ravel J Gajer P Abdo Z Schneider GM Koenig SS McCulle SL . Vaginal microbiome of reproductive-age women. Proc Natl Acad Sci USA. (2011) 108 Suppl 1:4680–7. doi: 10.1073/pnas.1002611107 20534435 PMC3063603

[40] Clifford GM Tully S Franceschi S . Carcinogenicity of human papillomavirus (HPV) types in HIV-positive women: a meta-analysis from HPV infection to cervical cancer. Clin Infect Dis. (2017) 64:1228–35. doi: 10.1093/cid/cix135 28199532 PMC5399941

[41] Kuller LH Tracy R Belloso W De Wit S Drummond F Lane HC . Inflammatory and coagulation biomarkers and mortality in patients with HIV infection. PloS Med. (2008) 5:e203. doi: 10.1371/journal.pmed.0050203 18942885 PMC2570418

[42] Dryden-Peterson S BvochoraNsingo M Suneja G Efstathiou JA Grover S Chiyapo S . HIV infection and survival among women with cervical cancer. J Clin Oncol. (2016) 34:3749–57. doi: 10.1200/JCO.2016.67.9613 27573661 PMC5477924

[43] Massad LS Xie X Burk RD D'Souza G Darragh TM Minkoff H . Association of cervical precancer with human papillomavirus types other than 16 among HIV co-infected women. Am J Obstet Gynecol. (2016) 214:354.e1–354.e3546. doi: 10.1016/j.ajog.2015.09.086 26433170 PMC4775397

[44] Kojic EM Kang M Cespedes MS Umbleja T Godfrey C Allen RT . Immunogenicity and safety of the quadrivalent human papillomavirus vaccine in HIV-1-infected women. Clin Infect Dis. (2014) 59:127–35. doi: 10.1093/cid/ciu238 24723284 PMC4305143

[45] Kaul R Prodger J Joag V Shannon B Yegorov S Galiwango R . Inflammation and HIV transmission in Sub-Saharan Africa. Curr HIV/AIDS Rep. (2015) 12:216–22. doi: 10.1007/s11904-015-0269-5 25877253

[46] Anorlu RI . Cervical cancer: the sub-Saharan African perspective. Reprod Health Matters. (2008) 16:41–9. doi: 10.1016/S0968-8080(08)32415-X 19027621

[47] Raymond AD CampbellSims TC Khan M Lang M Huang MB Bond VC . HIV type 1 Nef is released from infected cells in CD45(+) microvesicles and is present in the plasma of HIV-infected individuals. AIDS Res Hum Retroviruses. (2011) 27:167–78. doi: 10.1089/aid.2009.0170 20964480 PMC3064529

[48] Lee JH Schierer S Blume K Dindorf J Wittki S Xiang W . HIV-Nef and ADAM17-containing plasma extracellular vesicles induce and correlate with immune pathogenesis in chronic HIV infection. EBioMedicine. (2016) 6:103–13. doi: 10.1016/j.ebiom.2016.03.004 27211553 PMC4856776

[49] Vernon SD Hart CE Reeves WC Icenogle JP . The HIV-1 tat protein enhances E2-dependent human papillomavirus 16 transcription. Virus Res. (1993) 27:133–45. doi: 10.1016/0168-1702(93)90077-z 8384765

[50] Kim RH Yochim JM Kang MK Shin KH Christensen R Park NH . HIV-1 Tat enhances replicative potential of human oral keratinocytes harboring HPV-16 genome. Int J Oncol. (2008) 33:777–82. doi: 10.3892/ijo_00000064 18813791

[51] Noman MZ Hasmim M Messai Y Terry S Kieda C Janji B . Hypoxia: a key player in antitumor immune response. A review in the theme: cellular responses to hypoxia. Am J Physiol Cell Physiol. (2015) 309:C569–79. doi: 10.1152/ajpcell.00207.2015 26310815 PMC4628936

[52] Palmer CS Cherry CL Sada-Ovalle I Singh A Crowe SM . Glucose metabolism in T cells and monocytes: new perspectives in HIV pathogenesis. EBioMedicine. (2016) 6:31–41. doi: 10.1016/j.ebiom.2016.02.012 27211546 PMC4856752

[53] Mascanfroni ID Takenaka MC Yeste A Patel B Wu Y Kenison JE . Metabolic control of type 1 regulatory T cell differentiation by AHR and HIF1-α. Nat Med. (2015) 21:638–46. doi: 10.1038/nm.3868 26005855 PMC4476246

[54] Hegedus A Kavanagh Williamson M Huthoff H . HIV-1 pathogenicity and virion production are dependent on the metabolic phenotype of activated CD4+ T cells. Retrovirology. (2014) 11:98. doi: 10.1186/s12977-014-0098-4 25421745 PMC4252996

[55] Heller C Weisser T MuellerSchickert A Rufer E Hoh A Leonhardt RM . Identification of key amino acid residues that determine the ability of high risk HPV16-E7 to dysregulate major histocompatibility complex class I expression. J Biol Chem. (2011) 286:10983–97. doi: 10.1074/jbc.M110.199190 21321113 PMC3064153

[56] Chettimada S Lorenz DR Misra V Dillon ST Reeves RK Manickam C . Exosome markers associated with immune activation and oxidative stress in HIV patients on antiretroviral therapy. Sci Rep. (2018) 8:7227. doi: 10.1038/s41598-018-25515-4 29740045 PMC5940833

[57] Bandera A De Benedetto I Bozzi G Gori A . Altered gut microbiome composition in HIV infection: causes, effects and potential intervention. Curr Opin HIV AIDS. (2018) 13:73–80. doi: 10.1097/COH.0000000000000429 29045252

[58] VujkovicCvijin I Sortino O Verheij E Sklar J Wit FW Kootstra NA . HIV-associated gut dysbiosis is independent of sexual practice and correlates with noncommunicable diseases. Nat Commun. (2020) 11:2448. doi: 10.1038/s41467-020-16222-8 32415070 PMC7228978

[59] Cosper PF Bradley S Luo L Kimple RJ . Biology of HPV mediated carcinogenesis and tumor progression. Semin Radiat Oncol. (2021) 31:265–73. doi: 10.1016/j.semradonc.2021.02.006 34455982 PMC8409095

[60] Ashrafi GH Haghshenas MR Marchetti B O'Brien PM Campo MS . E5 protein of human papillomavirus type 16 selectively downregulates surface HLA class I. Int J Cancer. (2005) 113:276–83. doi: 10.1002/ijc.20558 15386416

[61] StumptnerCuvelette P Morchoisne S Dugast M Le Gall S Raposo G Schwartz O . HIV-1 Nef impairs MHC class II antigen presentation and surface expression. Proc Natl Acad Sci USA. (2001) 98:12144–9. doi: 10.1073/pnas.221256498 11593029 PMC59782

[62] Hunt PW Lee SA Siedner MJ . Immunologic biomarkers, morbidity, and mortality in treated HIV infection. J Infect Dis. (2016) 214 Suppl 2:S44–50. doi: 10.1093/infdis/jiw275 27625430 PMC5021241

[63] Doitsh G Galloway NL Geng X Yang Z Monroe KM Zepeda O . Cell death by pyroptosis drives CD4 T-cell depletion in HIV-1 infection. Nature. (2014) 505:509–14. doi: 10.1038/nature12940 24356306 PMC4047036

[64] Schwartz O Maréchal V Le Gall S Lemonnier F Heard JM . Endocytosis of major histocompatibility complex class I molecules is induced by the HIV-1 Nef protein. Nat Med. (1996) 2:338–42. doi: 10.1038/nm0396-338 8612235

[65] Punt S HouwingDuistermaat JJ Schulkens IA Thijssen VL Osse EM de Kroon CD . Correlations between immune response and vascularization qRT-PCR gene expression clusters in squamous cervical cancer. Mol Cancer. (2015) 14:71. doi: 10.1186/s12943-015-0350-0 25889974 PMC4392729

[66] Solomon B Young RJ Bressel M Cernelc J Savas P Liu H . Identification of an excellent prognosis subset of human papillomavirus-associated oropharyngeal cancer patients by quantification of intratumoral CD103+ immune cell abundance. Ann Oncol. (2019) 30:1638–46. doi: 10.1093/annonc/mdz271 31400196

[67] Komdeur FL Prins TM van de Wall S Plat A Wisman GBA Hollema H . CD103+ tumor-infiltrating lymphocytes are tumor-reactive intraepithelial CD8+ T cells associated with prognostic benefit and therapy response in cervical cancer. Oncoimmunology. (2017) 6:e1338230. doi: 10.1080/2162402X.2017.1338230 28932636 PMC5599086

[68] Bottley G Watherston OG Hiew YL Norrild B Cook GP Blair GE . High-risk human papillomavirus E7 expression reduces cell-surface MHC class I molecules and increases susceptibility to natural killer cells. Oncogene. (2008) 27:1794–9. doi: 10.1038/sj.onc.1210798 17828295

[69] Heeren AM Punt S Bleeker MC Gaarenstroom KN van der Velden J Kenter GG . Prognostic effect of different PD-L1 expression patterns in squamous cell carcinoma and adenocarcinoma of the cervix. Mod Pathol. (2016) 29:753–63. doi: 10.1038/modpathol.2016.64 27056074 PMC4931542

[70] Sahai E Astsaturov I Cukierman E DeNardo DG Egeblad M Evans RM . A framework for advancing our understanding of cancer-associated fibroblasts. Nat Rev Cancer. (2020) 20:174–86. doi: 10.1038/s41568-019-0238-1 31980749 PMC7046529

[71] Mariathasan S Turley SJ Nickles D Castiglioni A Yuen K Wang Y . TGFβ attenuates tumour response to PD-L1 blockade by contributing to exclusion of T cells. Nature. (2018) 554:544–8. doi: 10.1038/nature25501 29443960 PMC6028240

[72] Flach S Maniam P Hey SY Manickavasagam J . The molecular characteristics of recurrent/metastatic HPV-positive head and neck squamous cell carcinoma: A systematic review of the literature. Clin Otolaryngol. (2024) 49:384–403. doi: 10.1111/coa.14161 38658385

[73] Borgdorff H Tsivtsivadze E Verhelst R Marzorati M Jurriaans S Ndayisaba GF . Lactobacillus-dominated cervicovaginal microbiota associated with reduced HIV/STI prevalence and genital HIV viral load in African women. ISME J. (2014) 8:1781–93. doi: 10.1038/ismej.2014.26 24599071 PMC4139719

[74] Brotman RM Shardell MD Gajer P Tracy JK Zenilman JM Ravel J . Interplay between the temporal dynamics of the vaginal microbiota and human papillomavirus detection. J Infect Dis. (2014) 210:1723–33. doi: 10.1093/infdis/jiu330 24943724 PMC4296189

[75] Norenhag J Du J Olovsson M Verstraelen H Engstrand L Brusselaers N . The vaginal microbiota, human papillomavirus and cervical dysplasia: a systematic review and network meta-analysis. BJOG. (2020) 127:171–80. doi: 10.1111/1471-0528.15854 31237400

[76] Tugizov SM Herrera R ChinHong P Veluppillai P Greenspan D Michael Berry J . HIV-associated disruption of mucosal epithelium facilitates paracellular penetration by human papillomavirus. Virology. (2013) 446:378–88. doi: 10.1016/j.virol.2013.08.018 24074602 PMC3809142

[77] Gosmann C Anahtar MN Handley SA Farcasanu M AbuAli G Bowman BA . Lactobacillus-deficient cervicovaginal bacterial communities are associated with increased HIV acquisition in young South African women. Immunity. (2017) 46:29–37. doi: 10.1016/j.immuni.2016.12.013 28087240 PMC5270628

[78] Klein C Gonzalez D Samwel K Kahesa C Mwaiselage J Aluthge N . Relationship between the cervical microbiome, HIV status, and precancerous lesions. mBio. (2019) 10:e02785-18. doi: 10.1128/mBio.02785-18 30782659 PMC6381280

[79] Blank CU Haining WN Held W Hogan PG Kallies A Lugli E . Defining 'T cell exhaustion'. Nat Rev Immunol. (2019) 19:665–74. doi: 10.1038/s41577-019-0221-9 31570879 PMC7286441

[80] McLane LM Abdel-Hakeem MS Wherry EJ . CD8 T cell exhaustion during chronic viral infection and cancer. Annu Rev Immunol. (2019) 37:457–95. doi: 10.1146/annurev-immunol-041015-055318 30676822

[81] Miller E Bhardwaj N . Dendritic cell dysregulation during HIV-1 infection. Immunol Rev. (2013) 254:170–89. doi: 10.1111/imr.12082 23772620 PMC5590719

[82] Tokunaga R Zhang W Naseem M Puccini A Berger MD Soni S . CXCL9, CXCL10, CXCL11/CXCR3 axis for immune activation - a target for novel cancer therapy. Cancer Treat Rev. (2018) 63:40–7. doi: 10.1016/j.ctrv.2017.11.007 29207310 PMC5801162

[83] Pan Y Yu Y Wang X Zhang T . Tumor-associated macrophages in tumor immunity. Front Immunol. (2020) 11:583084. doi: 10.3389/fimmu.2020.583084 33365025 PMC7751482

[84] Allard B Longhi MS Robson SC Stagg J . The ectonucleotidases CD39 and CD73: novel checkpoint inhibitor targets. Immunol Rev. (2017) 276:121–44. doi: 10.1111/imr.12528 28258700 PMC5338647

[85] Chevalier MF Weiss L . The split personality of regulatory T cells in HIV infection. Blood. (2013) 121:29–37. doi: 10.1182/blood-2012-07-409755 23043072

[86] Routy B Le Chatelier E Derosa L Duong CPM Alou MT Daillère R . Gut microbiome influences efficacy of PD-1-based immunotherapy against epithelial tumors. Science. (2018) 359:91–7. doi: 10.1126/science.aan3706 29097494

[87] Derosa L Routy B Desilets A Daillère R Terrisse S Kroemer G . Microbiota-centered interventions: The next breakthrough in immuno-oncology? Cancer Discov. (2021) 11:2396–412. doi: 10.1158/2159-8290.CD-21-0236 34400407

[88] GarciaDiaz A Shin DS Moreno BH Saco J EscuinOrdinas H Rodriguez GA . Interferon receptor signaling pathways regulating PD-L1 and PD-L2 expression. Cell Rep. (2017) 19:1189–201. doi: 10.1016/j.celrep.2017.04.031 28494868 PMC6420824

[89] Benci JL Xu B Qiu Y Wu TJ Dada H TwymanSaint Victor C . Tumor interferon signaling regulates a multigenic resistance program to immune checkpoint blockade. Cell. (2016) 167:1540–1554.e12. doi: 10.1016/j.cell.2016.11.022 27912061 PMC5385895

[90] Templeton AJ McNamara MG Šeruga B VeraBadillo FE Aneja P Ocaña A . Prognostic role of neutrophil-to-lymphocyte ratio in solid tumors: a systematic review and meta-analysis. J Natl Cancer Inst. (2014) 106:dju124. doi: 10.1093/jnci/dju124 24875653

[91] Scott AC Dündar F Zumbo P Chandran SS Klebanoff CA Shakiba M . TOX is a critical regulator of tumour-specific T cell differentiation. Nature. (2019) 571:270–4. doi: 10.1038/s41586-019-1324-y 31207604 PMC7698992

[92] Khan O Giles JR McDonald S Manne S Ngiow SF Patel KP . TOX transcriptionally and epigenetically programs CD8+ T cell exhaustion. Nature. (2019) 571:211–8. doi: 10.1038/s41586-019-1325-x 31207603 PMC6713202

[93] Galon J Bruni D . Approaches to treat immune hot, altered and cold tumours with combination immunotherapies. Nat Rev Drug Discov. (2019) 18:197–218. doi: 10.1038/s41573-018-0007-y 30610226

[94] Chen DS Mellman I . Elements of cancer immunity and the cancer-immune set point. Nature. (2017) 541:321–30. doi: 10.1038/nature21349 28102259

[95] Borst J Ahrends T Bąbała N Melief CJM Kastenmüller W . CD4+ T cell help in cancer immunology and immunotherapy. Nat Rev Immunol. (2018) 18:635–47. doi: 10.1038/s41577-018-0044-0 30057419

[96] Morad G Helmink BA Sharma P Wargo JA . Hallmarks of response, resistance, and toxicity to immune checkpoint blockade. Cell. (2022) 185:576. doi: 10.1016/j.cell.2022.01.008 35120665

[97] Baruch EN Youngster I BenBetzalel G Ortenberg R Lahat A Katz L . Fecal microbiota transplant promotes response in immunotherapy-refractory melanoma patients. Science. (2021) 371:602–9. doi: 10.1126/science.abb5920 33303685

[98] Davar D Dzutsev AK McCulloch JA Rodrigues RR Chauvin JM Morrison RM . Fecal microbiota transplant overcomes resistance to anti-PD-1 therapy in melanoma patients. Science. (2021) 371:595–602. doi: 10.1126/science.abf3363 33542131 PMC8097968

[99] Hailemichael Y Johnson DH AbdelWahab N Foo WC Bentebibel SE Daher M . Interleukin-6 blockade abrogates immunotherapy toxicity and promotes tumor immunity. Cancer Cell. (2022) 40:509–523.e6. doi: 10.1016/j.ccell.2022.04.004 35537412 PMC9221568

[100] Branch C Parson-Martinez J Cory TJ . Drug-drug interactions in HIV-infected patients receiving chemotherapy. Expert Opin Drug Metab Toxicol. (2025) 21:15–27. doi: 10.1080/17425255.2024.2408004 39305240

[101] Chera BS Kumar S Shen C Amdur R Dagan R Green R . Plasma circulating tumor HPV DNA for the surveillance of cancer recurrence in HPV-associated oropharyngeal cancer. J Clin Oncol. (2020) 38:1050–8. doi: 10.1200/JCO.19.02444 32017652 PMC7106982

[102] Jeannot E Becette V Campitelli M Calméjane MA Lappartient E Ruff E . Circulating human papillomavirus DNA detected using droplet digital PCR in the serum of patients diagnosed with early stage human papillomavirus-associated invasive carcinoma. J Pathol Clin Res. (2016) 2:201–9. doi: 10.1002/cjp2.47 27917295 PMC5129558

[103] Thommen DS Koelzer VH Herzig P Roller A Trefny M Dimeloe S . A transcriptionally and functionally distinct PD-1+ CD8+ T cell pool with predictive potential in non-small-cell lung cancer treated with PD-1 blockade. Nat Med. (2018) 24:994–1004. doi: 10.1038/s41591-018-0057-z 29892065 PMC6110381

[104] Valpione S Mundra PA Galvani E Campana LG Lorigan P De Rosa F . The T cell receptor repertoire of tumor infiltrating T cells is predictive and prognostic for cancer survival. Nat Commun. (2021) 12:4098. doi: 10.1038/s41467-021-24343-x 34215730 PMC8253860

[105] Chowell D Morris LGT Grigg CM Weber JK Samstein RM Makarov V . Patient HLA class I genotype influences cancer response to checkpoint blockade immunotherapy. Science. (2018) 359:582–7. doi: 10.1126/science.aao4572 29217585 PMC6057471

[106] Platten M von Knebel Doeberitz N Oezen I Wick W Ochs K . Cancer immunotherapy by targeting IDO1/TDO and their downstream effectors. Front Immunol. (2015) 5:673. doi: 10.3389/fimmu.2014.00673 25628622 PMC4290671

[107] Marx V . Method of the year: spatially resolved transcriptomics. Nat Methods. (2021) 18:9–14. doi: 10.1038/s41592-020-01033-y 33408395

[108] Li Y Qiu H Zhao Z Qi F Cai P . Single-cell technologies and spatial transcriptomics: decoding immune low - response states in endometrial cancer. Front Immunol. (2025) 16:1636483. doi: 10.3389/fimmu.2025.1636483 40672944 PMC12263668

[109] Kiruba B Raja Karthikeyan RRR Anilkumar SR Sundararajan V Lulu SS . Implications of single-cell RNA sequencing in cervical cancer: Unravelling the molecular landscape. ACS Omega. (2025) 10:60080–93. doi: 10.1021/acsomega.5c06987 41427221 PMC12713431

[110] Jin R Zhang L . AI applications in HIV research: advances and future directions. Front Microbiol. (2025) 16:1541942. doi: 10.3389/fmicb.2025.1541942 40051479 PMC11882587

[111] Stripecke R Münz C Schuringa JJ Bissig KD Soper B Meeham T . Innovations, challenges, and minimal information for standardization of humanized mice. EMBO Mol Med. (2020) 12:e8662. doi: 10.15252/emmm.201708662 32578942 PMC7338801

[112] Lõhmussaar K Oka R Espejo ValleInclan J Smits MHH Wardak H Korving J . Patient-derived organoids model cervical tissue dynamics and viral oncogenesis in cervical cancer. Cell Stem Cell. (2021) 28:1380–1396.e6. doi: 10.1016/j.stem.2021.03.012 33852917

[113] Mahajan G Doherty E To T Sutherland A Grant J Junaid A . Vaginal microbiome-host interactions modeled in a human vagina-on-a-chip. Microbiome. (2022) 10:201. doi: 10.1186/s40168-022-01400-1 36434666 PMC9701078

