# Supplementary material for: HIV-associated immune remodeling in cervical cancer: implications for immunotherapy, biomarkers, and trial design
Source: Front Immunol. 2026 Sep 14;17:1953030. doi: 10.3389/fimmu.2026.1953030 (PMC13617522; doi:10.3389/fimmu.2026.1953030)
Supplement: Supplementary file 1 [file SupplementaryFile1.docx]

**Supplementary Table S1. Comparison of conventional tumor immune phenotypes with the proposed IEM state**

| **Feature** | **Immune-inflamed phenotype** | **Immune-desert/excluded phenotype** | **Proposed IEM state** |
| --- | --- | --- | --- |
| **Immune infiltration** | Generally abundant | Sparse or excluded from tumor nests | Present but potentially heterogeneous or spatially disorganized |
| **Effector function** | Partly preserved | Limited by poor recruitment or tumor access | Constrained by exhaustion and impaired antigen presentation |
| **Inflammatory state** | Potentially productive antitumor inflammation | Low or ineffective immune activation | Chronic inflammation may coexist with weak tumor-specific immunity |
| **Regulatory compartment** | Variable | May contribute to immune exclusion | Proposed myeloid, Treg, and metabolic suppression |
| **Mucosal context** | Not included in the definition | Not included in the definition | Barrier disruption and dysbiosis are defining contextual features |
| **Checkpoint response** | May be more favorable | Frequently limited | Unknown and requires prospective validation in WLWH |
| **Validation status** | Established framework | Established framework | Hypothesis-generating framework |

Note: The proposed IEM state is intended to complement, rather than replace, conventional tumor immune classifications by incorporating HIV-associated immune dysfunction and mucosal disruption.

Abbreviations: HIV, human immunodeficiency virus; IEM, inflamed, exhausted, and mucosally disrupted; Treg, regulatory T cell; WLWH, women living with HIV.
