# Supplementary material for: HIV-associated immune remodeling in cervical cancer: implications for immunotherapy, biomarkers, and trial design
Source: Front Immunol. 2026 Sep 14;17:1953030. doi: 10.3389/fimmu.2026.1953030 (PMC13617522; doi:10.3389/fimmu.2026.1953030)
Supplement: Supplementary file 2 [file SupplementaryFile2.docx]

**Supplementary Table S2. Candidate mechanism-matched strategies within the proposed IEM framework**

| **Target domain** | **Candidate strategies** | **Mechanistic rationale** | **Key consideration** |
| --- | --- | --- | --- |
| **Compounded T-cell exhaustion** | ICI combinations; therapeutic HPV vaccines | Restore or expand functional HPV-specific T-cell responses | Efficacy and toxicity require validation in WLWH |
| **Antigen-presentation impairment** | STING agonists; dendritic-cell-directed approaches; epigenetic modulation | Improve innate sensing, cross-presentation, and HLA-related antigen processing | Predominantly investigational, with limited HIV-specific evidence |
| **Myeloid-regulatory skewing** | CSF1R, CD39/CD73, or other myeloid-regulatory interventions | Reduce macrophage-, adenosine-, or Treg-associated suppression | Infection and immune-toxicity risks require evaluation |
| **Mucosal disruption and dysbiosis** | Defined live biotherapeutics or other microbiome-directed interventions | Restore mucosal ecology and reduce dysbiosis-associated inflammation | Clinical benefit in cervical cancer has not been demonstrated |
| **Chronic bystander inflammation** | Selective cytokine or inflammatory-pathway modulation | Reduce maladaptive inflammation while preserving antitumor immunity | JAK–STAT or cytokine blockade could also impair antiviral and antitumor responses |
| **Foundational HIV management** | ART optimization and management of active infection | Support viral control and immune recovery during cancer therapy | Remains standard supportive care rather than an IEM-specific treatment |

Note: The listed strategies are hypothesis-generating candidates for preclinical or clinical investigation and should not be interpreted as established treatments for HIV-associated cervical cancer.

Abbreviations: ART, antiretroviral therapy; CSF1R, colony-stimulating factor 1 receptor; HLA, human leukocyte antigen; HPV, human papillomavirus; ICI, immune checkpoint inhibitor; JAK–STAT, Janus kinase–signal transducer and activator of transcription; STING, stimulator of interferon genes; Treg, regulatory T cell; WLWH, women living with HIV.
