# Supplementary material for: HIV-associated immune remodeling in cervical cancer: implications for immunotherapy, biomarkers, and trial design
Source: Front Immunol. 2026 Sep 14;17:1953030. doi: 10.3389/fimmu.2026.1953030 (PMC13617522; doi:10.3389/fimmu.2026.1953030)
Supplement: Supplementary file 3 [file SupplementaryFile3.docx]

**Supplementary Table S3. Illustrative research stratification for HIV-inclusive cervical cancer immunotherapy studies**

| **Research group** | **Illustrative characteristics** | **Trial consideration** | **Suggested monitoring** |
| --- | --- | --- | --- |
| **A. Virologically suppressed with preserved immune function** | Suppressed HIV RNA, stable ART, and relatively preserved CD4⁺ count | Generally eligible for standard ICI trials if other criteria are met | HIV RNA, CD4⁺ count, routine efficacy and toxicity assessment |
| **B. Virologically controlled with incomplete immune recovery** | Suppressed or low-level HIV RNA with moderate CD4⁺ depletion | Should not be automatically excluded; particularly relevant to HIV-enriched prospective studies | More frequent virological, immunological, infectious, and inflammatory monitoring |
| **C. Uncontrolled or clinically unstable HIV disease** | Uncontrolled viremia, advanced CD4⁺ depletion, or active opportunistic infection | Requires individualized multidisciplinary assessment; prospective ICI safety data remain limited | Close HIV, infection, organ-function, and treatment-toxicity surveillance |

Note: These groups are proposed for research stratification rather than clinical treatment selection. CD4⁺ counts should not be interpreted as validated eligibility or treatment thresholds.

Abbreviations: ART, antiretroviral therapy; CD4, cluster of differentiation 4; HIV, human immunodeficiency virus; ICI, immune checkpoint inhibitor; RNA, ribonucleic acid.
